# Proteomic Biomarkers for Ageing the Mosquito *Aedes aegypti* to Determine Risk of Pathogen Transmission

**DOI:** 10.1371/journal.pone.0058656

**Published:** 2013-03-11

**Authors:** Leon E. Hugo, James Monkman, Keyur A. Dave, Leesa F. Wockner, Geoff W. Birrell, Emma L. Norris, Vivian J. Kienzle, Maggy T. Sikulu, Peter A. Ryan, Jeffery J. Gorman, Brian H. Kay

**Affiliations:** 1 Mosquito Control Laboratory, Queensland Institute of Medical Research, Brisbane, Queensland, Australia; 2 Protein Discovery Centre, Queensland Institute of Medical Research, Brisbane, Queensland, Australia; 3 Statistics Unit, Queensland Institute of Medical Research, Brisbane, Queensland, Australia; 4 Australian Army Malaria Institute, Brisbane, Queensland, Australia; 5 School of Environment, Griffith University, Brisbane, Queensland, Australia; 6 School of Biological Sciences, Monash University, Melbourne, Victoria, Australia; Universidade Federal do Rio de Janeiro, Brazil

## Abstract

Biomarkers of the age of mosquitoes are required to determine the risk of transmission of various pathogens as each pathogen undergoes a period of extrinsic incubation in the mosquito host. Using the 2-D Difference Gel Electrophoresis (2-D DIGE) procedure, we investigated the abundance of up to 898 proteins from the Yellow Fever and dengue virus vector, *Aedes aegypti*, during ageing. By applying a mixed-effects model of protein expression, we identified five common patterns of abundance change during ageing and demonstrated an age-related decrease in variance for four of these. This supported a search for specific proteins with abundance changes that remain tightly associated with ageing for use as ageing biomarkers. Using MALDI-TOF/TOF mass spectrometry we identified ten candidate proteins that satisfied strict biomarker discovery criteria (identified in two out of three multivariate analysis procedures and in two cohorts of mosquitoes). We validated the abundances of the four most suitable candidates (Actin depolymerising factor; ADF, Eukaryotic initiation factor 5A; eIF5A, insect cuticle protein Q17LN8, and Anterior fat body protein; AFP) using semi-quantitative Western analysis of individual mosquitoes of six ages. The redox-response protein Manganese superoxide dismutase (SOD2) and electron shuttling protein Electron transfer oxidoreductase (ETO) were subject to post-translational modifications affecting their charge states with potential effects on function. For the four candidates we show remarkably consistent decreases in abundance during ageing, validating initial selections. In particular, the abundance of AFP is an ideal biomarker candidate for whether a female mosquito has lived long enough to be capable of dengue virus transmission. We have demonstrated proteins to be a suitable class of ageing biomarkers in mosquitoes and have identified candidates for epidemiological studies of dengue and the evaluation of new disease reduction projects targeting mosquito longevity.

## Introduction

Mosquito-borne disease remains a persistent cause of global mortality and morbidity well into the twenty first century. There were approximately 3.3 billion people at risk and 655,000 reported malaria deaths in 2010 [1]; however a recent report suggests these are substantial underestimates [2]. Dengue is a worsening global pandemic resulting from infection from four related Flavivirus serotypes. Approximately 2.5 billion people are at risk with 50–100 million infections occurring each year [3] and the situation is worsening due to rapid urbanization and crowding, global trade and tourism and inefficient mosquito control [4]. Furthermore, an explosive outbreak of Chikungunya virus infection in the Indian Ocean islands demonstrated the potential for arboviruses to re-emerge as more virulent strains [5]. Mosquito longevity is a critical factor affecting mosquito-borne pathogens transmission cycles. The pathogens require time to proliferate within the mosquito and infect the salivary glands before they can be transmitted during a subsequent bite. Depending on environmental conditions, a period of 9–14 days is required for malaria parasites [6] and between 7–12 days for the dengue viruses [7]. Determination of mosquito population age structure therefore is important to assessing risk in the epidemiology of mosquito-borne disease [8] particularly as new strategies are targeting mosquito lifespan as a means of control [9], [10], [11].

A practical test for assessing mosquito age has been lacking due to an absence of obvious external age related changes. For several decades, predicting mosquito age required delicate dissections of ovaries to observe anatomical changes occurring during the reproductive history of females as a proxy measure for age [12]. The discovery of molecular biomarkers of ageing in insects has led to the development of more widely accessible age prediction assays but these may be cumbersome and expensive. The most promising age prediction assays for mosquitoes have measured age responsive changes to cuticular hydrocarbons separated by gas chromatography [13], gene transcriptional profiles measured using quantitative RT-PCR [14], [15] and near infrared (NIR) spectra instantly collected from cuticle scans [16]. The accuracy of these methods is variable; however, all require non-standard laboratory tests that require considerable initial or ongoing expenditure.

One potential solution to the problem is indicated from model organism studies in gerontology, when 25 years ago, Fleming and colleagues [17] demonstrated that significant alterations in protein expression occurred with age of *Drosophila melanogaster.* Proteins were visualized using two-dimensional (2-D) gel electrophoresis, separating proteins according to isoelectric point (pI) in one dimension and molecular weight (MW) in an orthogonal dimension [18]. Age related protein expression profiles could fit the criteria of “ageing biomarkers”; consistent age-related changes that could be used as surrogates for lifespan measurements in ageing research [19]. If protein biomarkers of ageing exist in mosquitoes, these could be measured using standard immunoassay techniques such as the Enzyme Linked Immunosorbant Assay (ELISA) which is available in most laboratories, including those in developing countries. Key to the use of proteins as biomarkers will be the reproducibility of the changes. Fleming’s studies on a sample of 100 *D. melanogaster* proteins indicated that ageing is associated with increased dysregulation of gene expression that could disrupt homeostasis and lead to cellular senescence. However, subsequent investigations of a more preliminary stage of gene expression, transcription, over the entire *D. melanogaster* genome identified characteristic age-related profiles but showed that the net biological variance over all genes remained stable [20]. For many genes, therefore, age related changes in expression are programmed and reproducible changes in protein abundance may follow accordingly.

In this investigation, we have applied 2-D DIGE analysis [21], a powerful improvement to the 2-D gel electrophoresis technique, to quantify changes to the *Ae. aegypti* proteome during ageing. We applied a mixed-effects model to examine variance in global protein expression during ageing to establish whether proteins are likely to be specific biomarkers of age, and selected specific candidates that matched robust biomarker discover criteria. We then validated the age related changes in abundance of these proteins in individual mosquitoes.

## Results and Discussion

**Figure 1:**
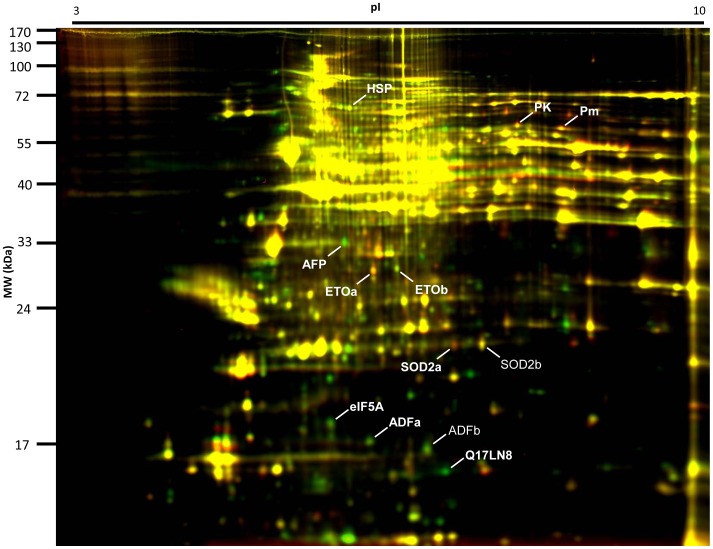
Differential proteome expression between two *Ae. aegypti* age samples (1 and 34 d old) compared using the 2-D DIGE procedure. Spots indicate individual proteins separated in one dimension according to the isoelectric point in an orthogonal dimension according to molecular mass. Spots appearing red represent proteins that are more highly expressed in the 34 d sample than the 1 d sample, whereas spots appearing green are more highly expressed in the 1 d sample. Yellow spots represent proteins that are equally abundant in the two age samples. Labeled spots indicate age-dependent proteins (bold) and related isoforms described in the text. Data supporting the identities shown and the identities of 86 other prominent spots are provided in Figure S4 and Table S3.

Protein lysates were obtained from *Ae. aegypti* females at three ages (1, 17 and 34 d) sampled from a recently colonized laboratory strain maintained under standard insectary conditions. Four replicates were collected at each age and the entire experiment was repeated on a second cohort of mosquitoes from the following generation. We produced 2-D DIGE gels of *Ae. aegypti* head and thorax proteins with proteins represented by discrete polypeptide spots in the pI 3–10 range. In each cohort, we defined 773 and 898 shared polypeptide spots for analysis. The 2-D proteomes of the age samples were compared in pairwise fashion, by false-coloring samples as either red (up-regulated) or green (down-regulated) with age (Figure 1). The normalized volume of each spot could be compared between all age samples within a cohort by following standard procedures for 2-D DIGE image analysis (including objective image warping and spot matching based on internal standard images).

We applied model fitting to describe the biological variation in protein quantity during ageing to assess the suitability of proteins as ageing biomarkers. First, we investigated whether protein abundance profiles clustered into common patterns over the three ages by fitting a mixed-effects model [22]. Abundance profiles fell into five significantly distinct clusters with the same result achieved for both cohorts (Figure S1). In both cases, two clusters defined decreasing abundance profiles of varying degrees, two described increasing abundance, and one cluster represented proteins with relatively stable profiles.

We then examined whether biological variance in protein abundance was equal or different between ages by specifying whether random effects were constant or heterogeneous between ages within the model. Likelihood ratio tests indicated that a heterogeneous specification significantly improved the model over a specification where random effects were held constant (Tables S1 and S2). This was observed from the significant reduction to the log likelihood of the heterogeneous random effects models for cohort one (χ^2^ = 360.1, df = 8, P<0.001) and cohort two (χ^2^ = 260.5, df = 8, P<0.001). Furthermore, the diagonal elements of the variance matrices decreased for all but one expression cluster. In other words, variance in protein abundance decreased between the three age groups from 1–34 d for four out of five abundance types.

This observation of decreasing variance in the abundances of proteins in older mosquitoes is in contrast with Fleming’s observations from *Drosophila* that ageing is associated with dysregulation of protein expression [17]. The reason for this discrepancy may be due to differing survival of the insects or methodological differences, such as the method of protein labeling (*in vivo* [^35^S]methionine labeling and *in vitro* minimal labeling of lysines with cyanine dyes). Assessments of *D. melanogaster* were carried out to an older age (44 d) than for *Ae. aegypti* (34 d), however increasing biological variance was reported between young and middle aged (28 d) *Drosophila* in contrast to our findings from mosquitoes. While the causes for these differences are unknown, decreasing variance in protein abundances over the ages examined provides support for their use as robust biomarkers of mosquito age over lifespans expected in field environments [23].

We selected proteins for further analysis if they were determined to be significantly associated with age in two out of three biomarker discovery analysis procedures. This rigorous approach was adopted to mitigate potential bias for selecting specific expression features that is inherent in each method. For each method, the Family Wise Error Rate (FWER) was controlled using the standard Bonferroni adjustment, ensuring that the probability of a type I error, that is the probability of at least one false positive, is less than or equal to α. Control of the FWER is a conservative approach recommended for biomarker discovery to prevent false positives carrying through to lengthy and expensive downstream procedures [24]. Furthermore, our final selection of candidates was of proteins meeting these criteria in two mosquito cohorts to mitigate possible environmental effects occurring during the maintenance of any one cohort. We found that 21.43–26.67% of proteins were shared between ANOVA and Limma analysis selections (Figure S2). Bootstrapping refined the Limma selections to the candidates with the strongest empirical support such that only 18.57–20.0% of selections were shared between ANOVA and Limma analysis. We refined our selection to 10 candidate spots that were selected by two out of three analyses in both cohorts (Figure S3).

**Figure 2:**
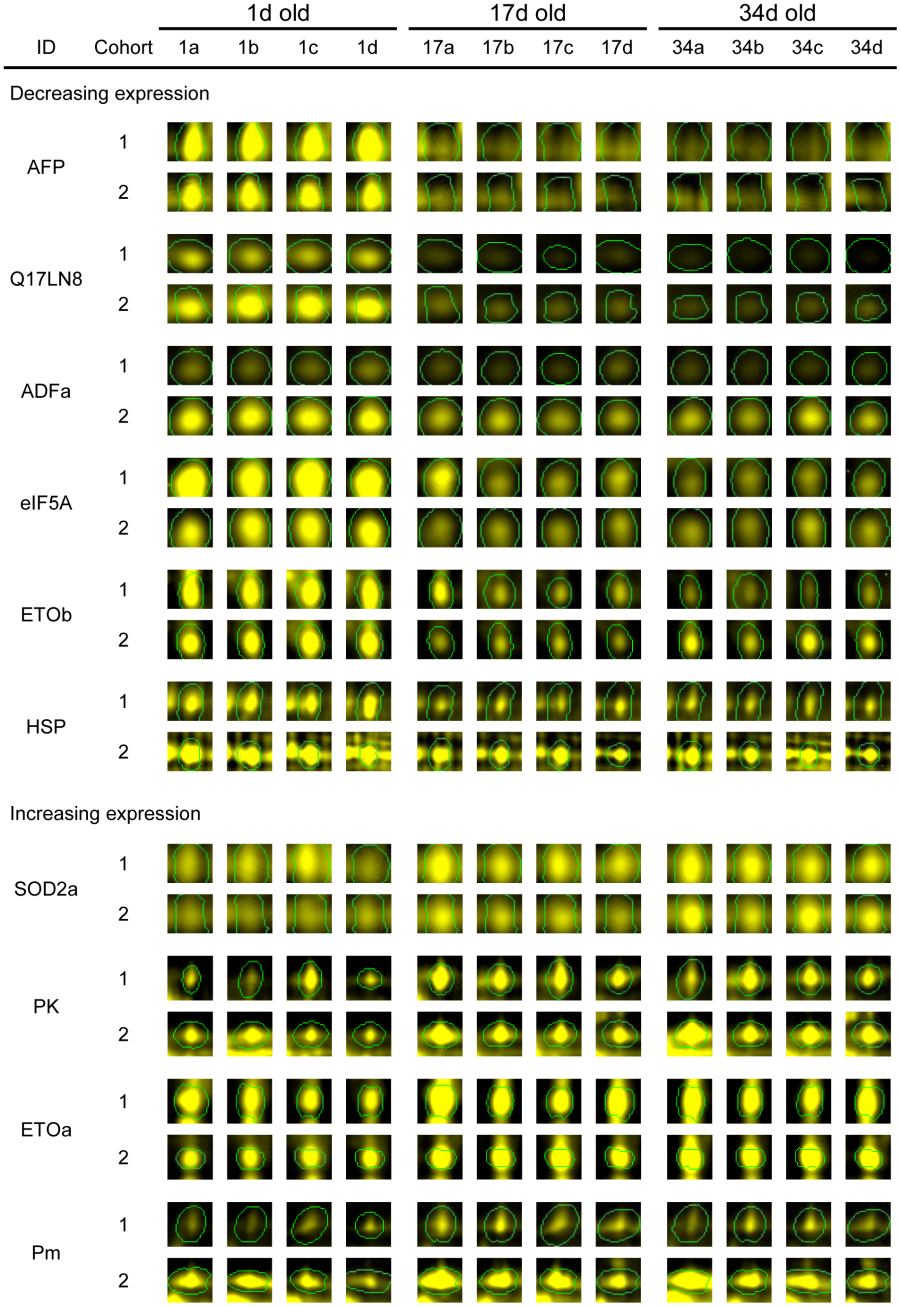
Spot album showing expression profiles of candidate *Ae. aegypti* protein age biomarkers. Profiles of spots are shown for three age samples (1, 17 and 34 d) and for four biological replicates at each age.

The 10 biomarker candidates and 86 other prominent spots were identified using in-gel trypsin digestion and Matrix Assisted Laser Desorption Ionization tandem Time of Flight (MALDI-TOF/TOF) mass spectrometry. Tandem mass spectra were searched against a database of *Ae. aegypti* protein sequences; proteins matching at least two peptides with Expectation (E)-values <0.05 were accepted as confident identifications (Figure S4 and Table S3). The most abundant proteins on the 2-D gels were associated with muscle (including actins, Myosin light chain 1 and 2 and Tropomyosin); probably reflecting a high proportional composition of indirect flight muscle in the tissue investigated, proton transport (ATPase*β*), glycolysis (Enolase) and detoxification (Glutathione *S*-transferases). The ten candidate ageing biomarkers were identified to be Pyruvate kinase (PK), an isoform of Manganese superoxide dismutase (SOD2) labeled SOD2a for acidic isoform, two isoforms of Electron Transport Oxidoreductase (ETO); labeled ETOa for acidic and ETOb for basic, eukaryotic Initiation Factor 5A (eIF5A), Actin depolymerizing factor (ADF), Q17LN8, Anterior Fat body Protein (AFP), Paramyosin (Pm) and a protein of approximately 70 kDa identified by a single peptide matching *Ae. aegypti* heat shock proteins (Figure S5; labeled HSP). Spot images of all ten biomarker candidates across three ages from both cohorts are displayed in Figure 2. A common feature to most of the candidates was a large change between 1 and 17 d old and a more limited change between 17 and 34 d old. Abundance of four candidates increased with age (PK, ETOa, SOD2a and Pm), whereas six candidates decreased (ETOb, eIF5A, ADFa, Q17LN8, AFP and HSP).

**Figure 3:**
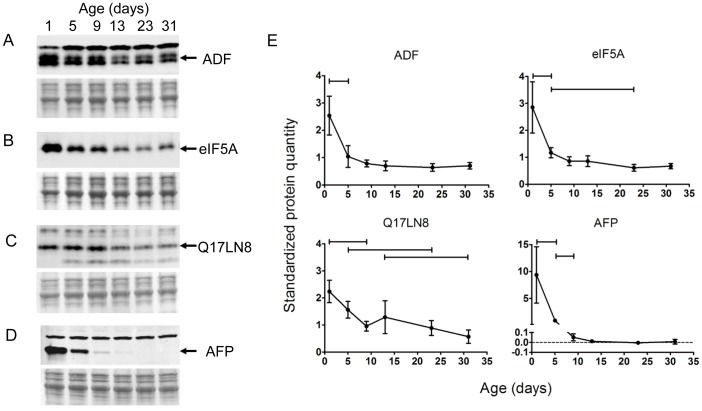
Differences in quantity of ageing biomarker proteins in individual *Ae. aegypti* females at various adult ages. Semi-quantitative western analysis was performed using polyclonal antibodies against the four ageing biomarker candidates; A. ADF, B. eIF5A, C. cuticle protein Q17LN8 and D. AFP. Below each panel is the corresponding Direct Blue 71-stained total-protein image from each membrane indicating consistent sample loading. E. Relative protein quantities determined by standardizing Western analysis bands (shown by arrows in A to D) by total protein and a gel standard sample. Values indicate the mean±SD standardized pixel volumes from five individual females at each age.

We validated the abundances of candidate biomarkers in *Ae. aegypti* females from a new cohort by semi-quantitative Western analysis. Three of the above proteins were known to be environmentally regulated (HSP, PK), or underwent low fold change (Pm) and therefore were unsuitable for further study. Investigation of the remainder required production of polyclonal antibodies against recombinant forms of the mosquito proteins (Table S4). Western analysis probing ADF, eIF5A, Q17LN8 and AFP expression in individual *Ae. aegypti* at six ages (1, 5, 9, 13, 23 and 31 d) showed decreasing abundance trends with age, confirming results from DIGE analysis (Figure 3). Significant changes were observed between 1 and 5 d for all four, and for some comparisons between older ages for candidates except ADF. Western analyses of pooled mosquitoes revealed no age-dependent change to SOD2 or ETO abundance from 1 to 34 d, probably because the polyclonal antibodies used recognized multiple pI isoforms concurrently (Figure S6). An increase in abundance of one pI isoform was matched by a decrease to the other.

There was an early age-related decline in the abundance of Actin depolymerization factor (ADF), which belongs to the ADF/cofilin family of proteins found in all eukaryotic cells (Figure 3 A and E). The actin-binding proteins are major regulators of actin filament assembly and disassembly required during cytoskeletal remodeling in cellular processes such as motility, cell polarity during migration and chemotaxis [25], [26]. ADF/cofilin severs F-actin increasing the rate of actin polymerization, however it also binds filamentous actin increasing the rate of depolymerization. Activity of the protein is regulated by phosphorylation. The *D. melanogaster* homolog of ADF/cofilin; Twinstar, is also required for the regular orientation of hairs on fly wings [25]. High abundance of *Ae. aegypti* ADF at 1 d old could be a remnant of large-scale cytoskeletal remodeling occurring during metamorphosis.

The *Ae. aegypti* homolog of eukaryotic Initiation Factor 5A (eIF5A, formerly eIF-4B) showed a remarkable consistency in downward expression from 1–31 d between individual females. Expression was highest at 1d and dropped by approximately 3-fold between 1 and 5d of adult age with additional decreases over the lifespan (Figure 3 B and E). eIF5A is a highly conserved and essential protein required for cell proliferation and is one of only two proteins (with eIF5A2) containing the atypical amino acid hypusine [27]. The protein was first classified as an initiation factor due to its ability to enhance *in vitro* methionyl-puromycin synthesis, simulating synthesis of the first peptide bond [28], a role recently confirmed in yeast [29]. In *Drosophila*, eIF5A plays roles in larval growth and development, adult organ size and autophagy [30]. eIF5A is upregulated in a mosquito cell line (*Aedes albopictus-*derived C6/36 cells) in response to dengue infection [31]. Here, we report a decrease in eIF5A quantity during ageing with possible consequences for cellular translation.

Expression of a protein with high amino acid sequence similarity to insect cuticle proteins (Q17LN8) decreased by 2 fold between 1 and 31d (Figure 3 C and E). High expression of cuticle proteins early in adult lifespan may be attributed to the deposition of the endocuticle that occurs during maturation of very young adult mosquitoes [32]. We show a consistent decline in abundance of this protein; however the effect on the integrity of the cuticle is unknown. Insect cuticle provides a barrier against insecticide absorption, a trait under selective pressure in the development of insecticide resistance. Interestingly, mosquitoes from insecticide-resistant strains lose resistance as they age [33]. Correlated changes to mosquito cuticle may provide leads on potential resistance mechanisms.

The most distinct ageing related change was observed from AFP in young adult mosquitoes. AFP expression decreased by 10-fold in individual *Ae. aegypti* females from 1 to 5 d and then decreased by a further 10-fold to 9 d of age (Figure 3 D and E). At older ages, AFP expression had decreased to below the limit of sensitivity of Western analysis and could not be observed, except for very feint bands in two individuals. The amino acid sequence of *Ae. aegypti* AFP is highly similar to the anterior fat body proteins of other Diptera. The first insect homolog was described from the flesh fly *Sarcophaga perigrina* [34]. It was localized to granules within the *S. perigrina* fat body, the organ best known for its roles in insect nutrient storage and utilization, sharing some functional similarities with the mammalian liver [35]. A decrease in the abundance of AFP was correlated to disintegration of the anterior lobe of the fat body during pupation. Protein granules within fat body trophocytes of *Ae. aegypti* decrease in abundance and size between 1 and 18 d of adult age [36]. Immunohistochemical studies with specific AFP antibodies are required to determine whether these granules represent the location of AFP within the *Ae. aegypti* fat body. Little is known of the function of AFP. Due to strong interaction with receptors of hexamerin storage proteins, AFP could be involved with regulation of hexamerin endocytosis [37]. AFP was identified among *Drosophila* proteins upregulated in response to septic injury [38]. Interestingly, AFP shares up to 30% amino acid sequence similarity to mammalian Senescence Marker Protein-30 (SMP-30; regucalcin) family proteins. SMP-30 decreases during ageing in rat liver [39].

In addition to the identification of ageing biomarkers, we have provided insight into other ageing- related changes from the *Ae. aegypti* proteome. We show that ageing is associated with the changes in the relative quantity of pI isoforms of SOD2 and ETO (Figure 1). The presence of multiple pI isoforms of a protein on 2-D gels indicates the action of post-translation modification (PTM). PTMs including phosphorylation, acetylation of lysine and oxidation of cysteine are known to change protein charge state and pI [40]. Known PTMs of SOD2 include carbonylation, nitration, phosphorylation, S-glutathionylation [41], and lysine acetylation in a mechanism of enzyme regulation mediated by Surtuin-3 (Sirt3) in response to stress [42]. ETO shares conserved domains belonging to the Electron Transport Flavoprotein (ETF) superfamily and has high sequence similarity to invertebrate and vertebrate Electron Transport Flavoprotein subunit α proteins. ETF transfers electrons from the oxidative breakdown of fatty acids and catabolism of amino acids to the reduction of ubiquinone within the electron transport chain [43]. Lysine acetylation produces isoforms of mammalian ETF with varying charge states [44] and is a form of Sirt3-mediated regulation of energy homeostasis [45]. Here, we show ageing is associated with PTM(s) of these proteins but not protein abundance (Figure S6). Further research is required to define the modifications acting here and whether these are associated with age dependent regulation or loss of function of these essential enzymes.

Furthermore, three genes that show age-related changes in transcription in *Ae. aegypti* (*Calcium binding protein* [14], *Ubiquinol-cytochrome c reductase complex core protein* and *2-oxoglutarate dehydrogenase* [15]) were identified in our experiments but no significant age related changes in protein abundance were observed. For example, the calcium binding protein gene undergoes a highly consistent two-fold decrease in transcription [14], [15] but the protein was consistently expressed between 1 and 34 d (Spot 75 in Figure S4 and Table S3) here. These results echo recent findings in yeast of limited co-variation between transcription and protein levels across the genome [46] and demonstrate the need for proteomic assessment to validate ageing and other hypotheses based solely on transcriptional change.

By examining the changes in expression of proteins during ageing in *Ae. aegypti* we show that proteins can be biomarkers of ageing and have identified four specific candidates; ADF, eIF5A, Q17LN8 and AFP. Their decreased variance up to 34 d, in keeping with more general observations across the proteome; indicates that these candidates are likely to retain a tight association with ageing. In particular, AFP has the potential to be an ideal biomarker to predict dengue transmission risk. AFP decreases rapidly to below detectable levels in mosquitoes older than 13 d, approximately coinciding with the age at which *Ae. aegypti* could become capable of dengue virus transmission. This marker could be used to delineate a subpopulation of mosquitoes that are potentially infectious. By determining the identities of prominent spots on 2-D gels, we have established a valuable 2-D proteome map of this prominent mosquito vector (Figure S4 and Table S3). By validating the trends originally identified from 2-D DIGE analysis we confirm the power of this approach for defining ageing-dependent proteome change and biomarker discovery in *Ae. aegypti* and other organisms with sequenced genomes.

Future studies will determine the breadth of applicability of these markers to other *Ae. aegypti* strains and to other species; however, we can confirm that age dependent changes in abundance of the four biomarkers tested by Western analysis also occur in a Vietnamese strain of *Ae. aegypti*, with AFP and Q17LN8 providing the greatest discrimination between ages. The responses of these biomarkers to environmental regulation and infection with dengue viruses and microbial biological control agents, including *Wolbachia* endosymbiotic bacteria, should also be evaluated before the utility of these biomarkers can be fully established. If the trends observed here prove robust to these influences, these proteins are likely to serve as new biomarkers of mosquito age for epidemiological studies of dengue and to evaluate disease reduction projects that aim to reduce mosquito longevity.

## Materials and Methods

### Ethics Statement

Human research ethics approval for allowing colonized mosquitoes to feed on the investigators was obtained from the Queensland Institute of Medical Research Human Research Ethics Committee (HREC P1162). Blood feeding was considered to cause a medium risk of allergic reaction and provision was in place that individuals were excluded if they reacted strongly to bites. Written consent was obtained acknowledging the right to refuse or withdraw. No specific permits were required for the described field studies. The field studies did not involve protected or endangered species. Verbal consent was obtained from residents in north Queensland to set mosquito ovitraps and collect mosquito eggs from their property.

### Mosquitoes

*Aedes aegypti* mosquitoes were obtained from an F_10–11_ colony established from collections of eggs in the location of Cairns, Queensland, Australia. Ovitraps were set on >50 residential properties in the suburbs of Cairns. The colony was subsequently maintained at the Queensland Institute of Medical Research (QIMR) insectary (27°C, 70% humidity and 12∶12 hr day:night light cycling with 30 min crepuscular periods). To raise discrete cohorts of mosquitoes, eggs were vacuum-hatched and larvae were reared in groups of 50 in 500 ml aged tap water and fed on ground fish food pellets (TetraMin Tropical Tablets, The Rich Mix, Blacksburg, VA) *ad libitim*. Adult mosquitoes were maintained in cages and provided with a constant supply of 10% sucrose solution. Mosquitoes were blood-fed on the arm of a human volunteer at 3–4 d old and subsequently at 7d intervals. Adult females were harvested at 1, 17 and 34 d post emergence by aspirating a sample of females from the cages, anaesthetizing specimens using CO_2_, briefly immobilizing the mosquitoes on a chill table before snap freezing the specimens in liquid nitrogen. For DIGE experiments, age samples were collected from two cohorts of mosquitoes separated by one generation. For semi-quantitative Western analysis experiments, a third cohort of larvae were provided with TetraMin at 0.13, 0.25, 0.38 and 0.50 mg/larva/day for I, II, III and IV instars, respectively and adult females were sampled at 1, 5, 9, 13, 23 and 31 d post emergence.

### Protein Extraction and Preparation

Female mosquitoes were dissected on a bed of dry ice to isolate the heads and thoraces. Tissue from five females were pooled together in 2 ml screw cap vials containing a 3 mm silica glass bead and 100 µl of UTC buffer (7M Urea, 2M Thiourea and 4% (w/v) CHAPS) and homogenized by shaking the vials for 1.5 min using a MiniBeadbeater -96 (BioSpec Products, Bartlesville, OK, USA). Cuticular material was pelleted by centrifugation at 12,000 × g, 4°C for 5 min and 90 µl of supernatant was transferred to a new tube. Protein was precipitated and washed using the 2-D Clean-Up kit (GE Healthcare, Waukesha, WI, USA). Protein pellets were re-solubilized in 100 µl of 2-D lysis buffer (30 mM Tris pH 8.5, 7 M Urea, 2 M Thiourea, 4% (w/v) CHAPS). Protein quantity was measured from a sample of 2 µl using the 2-D Quant protein quantification kit (GE Healthcare). An internal protein standard for DIGE analysis was created by pooling 50 µg of protein from each age sample.

### 2-D Difference In-gel Electrophoresis (2-D DIGE)

Using the CyDye DIGE Fluor, minimal labeling kit (GE Healthcare), four samples at each of three ages (1, 17 and 34 d old) were labeled with 400 pmol of either Cy3 or Cy5 cyanine dyes and the internal standard labeled with Cy2 as described in [47]. Equal quantities (20 µl) of two age samples and the internal standard were pooled. We then added 5.4 µl DeStreak rehydration solution (GE Healthcare), 4.5 µl of BioLyte 3/10 100× ampholytes (Bio-Rad laboratories, Richmond, CA, USA) and 381 µl of UTC buffer. This was applied onto 24 cm pH 3–10 linear IPG ReadyStrips (Bio-Rad) and incubated for 1 hr at room temperature. The strips were overlaid with mineral oil and incubated overnight. The oil was drained and isoelectric focussing of proteins performed using a PROTEAN IEF Cell (Bio-Rad) at 250V for 15 min, a linear increase to 10,000 V in 3 hr, and at 10,000 V for a total of 70,000 V hr^−1^. IPG strips were equilibrated for 10 min in 8 ml of equilibration buffer I (6 M Urea, 0.375 M Tris-HCL pH 8.8, 2% [w/v] SDS, 20% glycerol and 2% [w/v] DTT) followed by 10 min in 8 ml of equilibration buffer II (6 M Urea, 0.375 M Tris-HCL pH 8.8, 2% [w/v] SDS, 20% glycerol and 2.5% [w/v] iodoacetamide). Equilibrated IPG strips were then placed on top of 12% acrylamide gels (250 × 205 × 1 mm) in optically clear plates which were then sealed with 1% (w/v) agarose in SDS running buffer and 0.3% bromophenol blue. Electrophoresis was performed at 5 mA per gel for 15 min, 10 mA per gel for 15 and then 30 mA per gel to completion. Labeled proteins were visualized by scanning each gel using a Typhoon 9400 (GE Healthcare) scanner at the appropriate excitation and emission wavelengths for the dyes (Cy2, 488 nm, Cy3 532 nm and Cy5, 633 nm) at 100 µm resolution. Analysis of proteins spots was performed using Delta 2D version 4.0 software (Decodon, Greifswald, Germany) as described in [47].

### Analysis of Changes in Global Variance in Protein Abundance with Age

Mixed effects models have been utilized to cluster genes and formulate tests of differential expression in DNA and mRNA microarray analyses, respectively [22]. The strength of these methods is that they capture interdependence not only between replicated measurements, but also within samples. We used this methodology in a new approach to make inferences about the overall variability of proteins in regards to increasing age of *Ae. aegypti*. Consider a vector that contains the measurements for protein *j* denoted y_j_. Conditional on the membership of the *j*th protein in the *h*th component of the mixture, the vector *y_j_* can be written
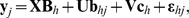
where 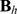 represents the coefficient of fixed effects for the *h*th cluster, 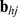 represents the random effects for the *h*th cluster, 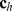 represents the random effects for the tissue samples in the *h*th cluster and 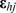 is the error for protein *j* in the *h*th cluster. **X**, **U**, and **V** are design matrices which are chosen to reflect the experimental design. This formulation allows proteins from the same sample in the same cluster to be correlated, which is a more realistic model for tissue samples. In addition, the random effects for the *h*th cluster can be defined to account for the heterogeneity of genes across experimental conditions, given that it exists.

We defined the design matrices such that, conditional of membership of the *h*th cluster, each protein at each age has a fixed effect and a random effect added to each protein expression level at each age. The random effect being normally distributed with mean and covariance matrix *σ_hs_^2^* ***I_s_*** where *σ_hs_^2^* is either allowed to vary between the *s* ages (heterogeneous version), or is restricted to be equal for all classes (homogeneous version). In addition, each tissue in each cluster was given a random effect which induces correlation amongst the proteins in the same tissue sample within the cluster. This random effect was also normally distributed with mean zero and covariance matrix *σ_h_^2^* ***I_m_***. The number of clusters fitted to the data was dictated by the Bayesian Information Criteria (BIC). When the model was fitted either the homogeneous or heterogeneous version was specified. The superiority of one specification over the other was determined via a likelihood ratio test.

### Ageing Biomarker Discovery

We employed a rigorous procedure to identify age dependent polypeptides which involved finding polypeptide spots that were identified as significantly age-associated by three statistical procedures (ANOVA with permutation provided by the Delta-2D package, the Limma procedure [48] provided for the R statistics environment and the Limma procedure applied to 1000 bootstrap samples of the data). For all analyses, α was set to 0.01 and the Bonferroni FWER correction was applied. Candidate biomarkers were chosen if they were found to be significantly associated with age by two out of the three analyses in both cohorts.

### Protein Identification

#### In-gel digestion

Preparative 2-D gels of *Ae. aegypti* proteins were produced for spot identification. Up to 800 µg of unlabelled *Ae. aegypti* head and thorax protein extract per gel was electrophoresed in the first dimension in 11 cm 3–10 IPG Ready strips and in the second dimension in 12% acrylamide gels (148 × 108 × 1 mm). Gels were stained using Colloidal Coomassie solution (0.12% [w/v] Coomassie G250, 10% [w/v] NH_4_SO_4_, 10% phosphoric acid, 20% methanol in distilled H_2_O). Two mm diameter gel plugs were manually excised from spots of interest and destained overnight in 300 µl of MS Fix solution (40% ethanol, 10% acetic acid in distilled H_2_O) on an orbital shaker. Replicate gel plugs of the same protein spot were excised from up to eight gels. The MS Fix was discarded and 100 µl of 25 mM ammonium bicarbonate, pH 8, was added to the samples which were incubated, shaking, for 15 min. The ammonium bicarbonate wash was repeated. The plugs were dried using a vacuum concentrator for 30 min. Four µl of 20 pg/µl trypsin (proteomics grade; Sigma Aldrich, Sigma-Aldrich, St Louis, MO, USA) in 40 mM ammonium bicarbonate/10% acetonitrile (Burdick and Jackson, Muskegon, MI, USA) and incubated at room temperature for 1 hr. A further 35 µl of ammonium bicarbonate/acetonitrile (minus trypsin) was added and the samples incubated overnight at 37°C. The fluid bathing corresponding replicate gel plugs was pooled. Twenty µl of 50% acetonitrile/0.1% trifluoroacetic acid (TFA) in distilled H_2_O was added to the gel plugs for one hour at room temperature, pooled with the previous extracts and the extracts were lyophilized.

#### MALDI-TOF/TOF Mass spectrometry

Peptides were purified using C-18 ZipTips (Millipore, Bedford, MA), combined with α-cyano-hydroxy-cinnamic acid (α-CHCA) matrix and MALDI-TOF/TOF mass spectrometry and protein database searching performed using procedures described in [47] with the following modifications. Either an Autoflex or Ultralflex III MALDI-TOF/TOF mass spectrometer (Bruker, Bremen, Germany) was used depending on sensitivity requirements for particular samples. We used a database containing UniProtKB sequences for the *Ae aegypti* taxonomy and common contaminants (bovine trypsin and human keratins). The database of 17208 sequences was downloaded from http://www.uniprot.org/on 9 January 2012. Fragment ion and parent ion mass tolerances were set to 0.8 Da and 100 ppm, respectively, for Ultraflex data and 1.0 Da and 150 ppm for Autoflex data. We specified a peptide acceptance criteria of an E-value <0.05 and required at least two accepted peptide identifications to assign a protein identification. Annotated spectra are provided as supporting information for proteins identified by a single peptide match.

### Recombinant Protein Expression

We prepared recombinant proteins of the candidate *Ae. aegypti* age biomarker proteins fused to Glutathione *S-*transferase (GST). We ligated cDNA covering the full length or partial transcripts of proteins into the plasmid pGEX-6P-1 (GE Healthcare) and confirmed that the transcripts and the GST tag were in frame by sequencing the constructs from dH5α *E. coli* cells. Suitable constructs were used to transform BL21 strain *E. coli*. We then induced expression of the recombinant protein and batch purified the protein using Glutathione Sepharose 4B (GE Healthcare) according to the methods of Frangioni and Neel [49].

### Antibody Production and Purification

The recombinant proteins were used as antigens for polyclonal antibody production in rabbits, sheep or goats by the Institute for Medical and Veterinary Science (IMVS), Adelaide, Australia. We purified the antibodies from the anti-sera using affinity chromatography with GST or the recombinant proteins crosslinked to Glutathione Sepharose 4B.

### Semi-quantitative Western Analysis

We performed semi-quantitative western analysis using the prepared mosquito specific polyclonal antibodies. We separated 5 µg of protein from head and thorax lysates of individual female mosquitoes from six ages (1, 5, 9, 13, 23 and 31 d) or from pools of five females for SOD2 and ETO proteins. Each lysate was loaded in duplicate on a 12% MiniProtean TGX gel (Bio-Rad) together with a gel standard. Protein was transferred to PVDF membrane (Bio-Rad) using a Transblot Turbo semi-dry transfer apparatus (Bio-Rad). Blotting was performed using the polyclonal primary antibodies according to parameters provided in Table S4. We used secondary antibodies coupled to infra-red (IR) fluorophores (Li-cor, Lincoln, NE) and visualized blots using a Licor Odyssey IR scanner. Total protein quantity was used as a loading control by post staining membranes with Direct Blue 71 stain (Sigma-Aldrich, St. Louis, MO) [50] and scanning membranes at 600 dpi with a bright field scanner (Epson, Suwa, Nagano, Japan). Western analysis band intensities were standardized by the combined pixel intensity of a region of Direct Blue 71 stained proteins of approximately 35–55 kDa (Figure 3) and the gel-standard. This MW range was chosen due to an absence of ageing-related changes among included proteins (Figure 1). 2-D gel electrophoresis for 2-D western analyses was performed using 11 cm pI 5-8 IPG strips (Bio-Rad) using procedures described above.

## Supporting Information

Figure S1 **Cluster diagrams of predominant protein expression profiles during aging in** ***Ae. aegypti*** **.** Five underlying clusters were identified from both cohorts by fitting the mixed effects model with increasing numbers of clusters and comparing BIC values. A. Clusters defined from cohort one. B. Clusters defined in experiment from cohort two. Each line represents the mean protein abundance over four replicates and is given a level of grey based on the rank of the posterior probability of membership in the *h*th cluster with black being the highest ranked protein and white being the lowest ranked protein.
(TIF)

Figure S2 **Venn diagrams depicting the relationships between three statistical procedures used to identify age-related differences in protein abundance.** The three procedures were ANOVA with permutation, Limma and the Limma procedure applied to 1000 bootstrap samples of the data. The procedures were applied to DIGE protein expression profiles from aged *Ae. aegypti* females from two experimental cohorts. A. Cohort one (n = 773). B. Cohort two (n = 898).
(TIF)

Figure S3 **Age dependent spots detected from two** ***Ae. aegypti*** **cohorts using strict criteria for biomarker discovery.** 2-D DIGE fusion images are shown for A. cohort one and B. cohort two. Circled spots indicate proteins determined to be significantly age-responsive in two out of three statistical analyses. Arrows indicate candidates shared between two mosquito cohorts.
(TIF)

Figure S4 **Two-dimensional gel electrophoresis map of the** ***Ae. aegypti*** **female head and thorax proteome showing spots identified by MALDI-TOF/TOF mass spectrometry.** Numbers correspond to the protein identities in Table S3.
(TIF)

Figure S5 **Annotated MS/MS spectra of a peptide from biomarker candidate HSP matching a conserved peptide from** ***Ae. aegypti*** **heat shock proteins.**
(TIF)

Figure S6 **Western analyses of SOD2 and ETO proteins in aged** ***Ae. aegypti*** **mosquitoes.** One dimensional western analysis of pools of five female *Ae. aegypti* using A. pan SOD2 and C. pan ETO antibodies demonstrated unchanging protein abundance from 1–34 d old. Below each panel is Direct Blue 71 stained total protein from each membrane indicating consistent sample loading. Two dimensional western analyses of one d old age samples using B. pan SOD2 and D. pan ETO polyclonal antibodies showed antibody recognition of potentially modified isoforms of the proteins, present as multiple protein spots or streaks on the horizontal (pI) axis.
(TIF)

Table S1 **Variance matrices for unrestricted and restricted mixed effects models fitted to cohort one for examining clustering and biological variance of protein expression profiles.**
(DOCX)

Table S2 **Variance matrices for heterogeneous and homogenous mixed effects models fitted to cohort two for examining clustering and biological variance of protein expression profiles.**
(DOCX)

Table S3 **Identities of proteins excised from preparative 2-D gels of female** ***Ae*** **.** ***aegypti*** **head and thorax tissue.** Location of the spots is provided in Figure S4. Protein identities were determined using in-gel tryptic digestion and MALDI-TOF/TOF mass spectrometry as described in the text. Proteins matching at least two peptides with E-values <0.05 were accepted as confident identifications. ‡ An annotated MS/MS spectra of a peptide from spot 10 matching *Ae. aegypti* heat shock proteins is provided in Figure S5.
(DOCX)

Table S4 **Custom prepared polyclonal antibodies and Western analysis conditions used to validate** ***Ae. aegypti*** **ageing biomarker proteins.**
(DOCX)
