# Supplementary figures and images for: Proteomic Biomarkers for Ageing the Mosquito Aedes aegypti to Determine Risk of Pathogen Transmission
Source: PLoS One. 2013 Mar 11;8(3):e58656. doi: 10.1371/journal.pone.0058656 (PMC3594161; doi:10.1371/journal.pone.0058656)

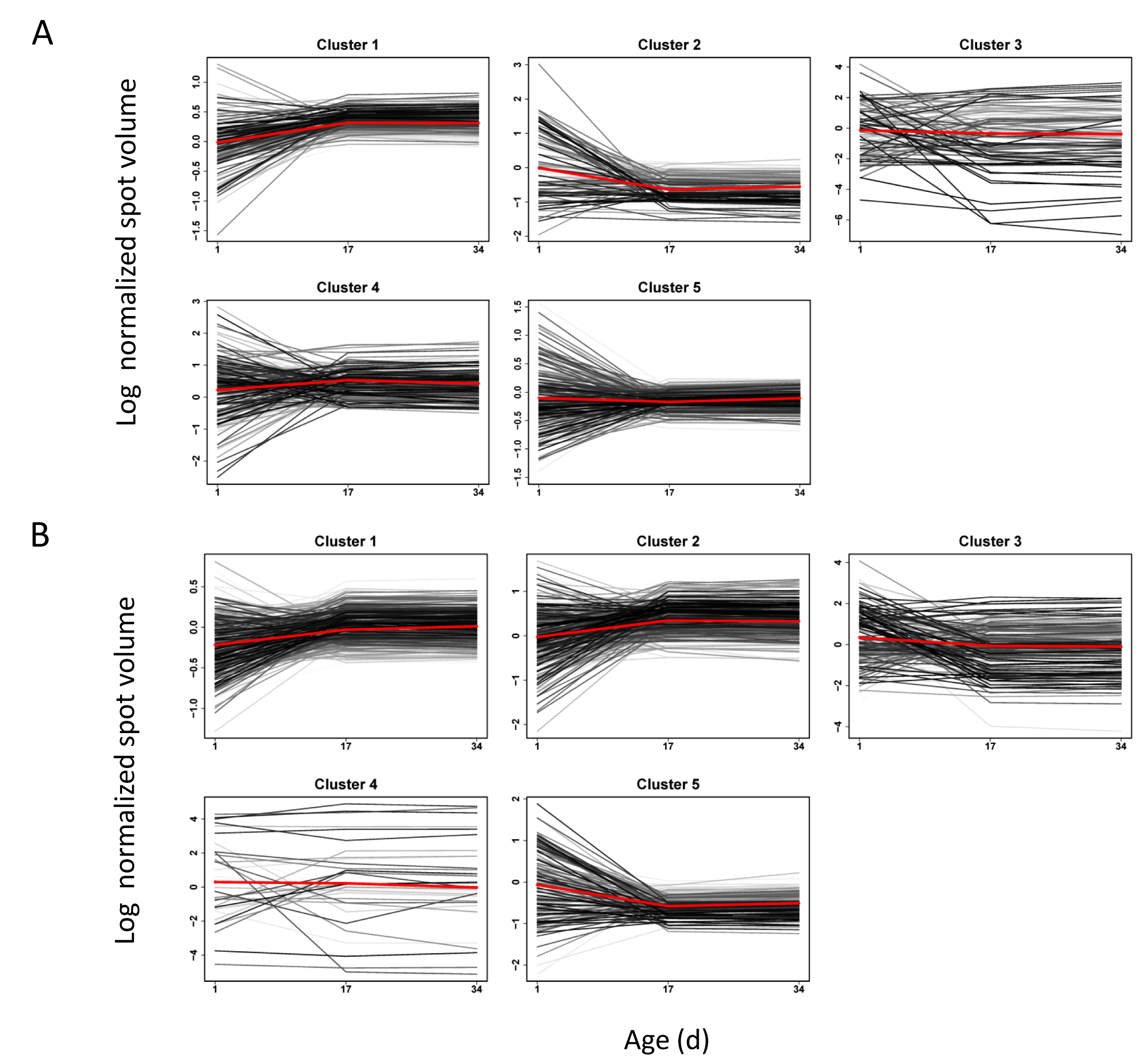

Supplement: Figure S1 — Cluster diagrams of predominant protein expression profiles during aging in Ae. aegypti . Five underlying clusters were identified from both cohorts by fitting the mixed effects model with increasing numbers of clusters and comparing BIC values. A. Clusters defined from cohort one. B. Clusters defined in experiment from cohort two. Each line represents the mean protein abundance over four replicates and is given a level of grey based on the rank of the posterior probability of membership in the hth cluster with black being the highest ranked protein and white being the lowest ranked protein. (TIF) [file pone.0058656.s001.tif]

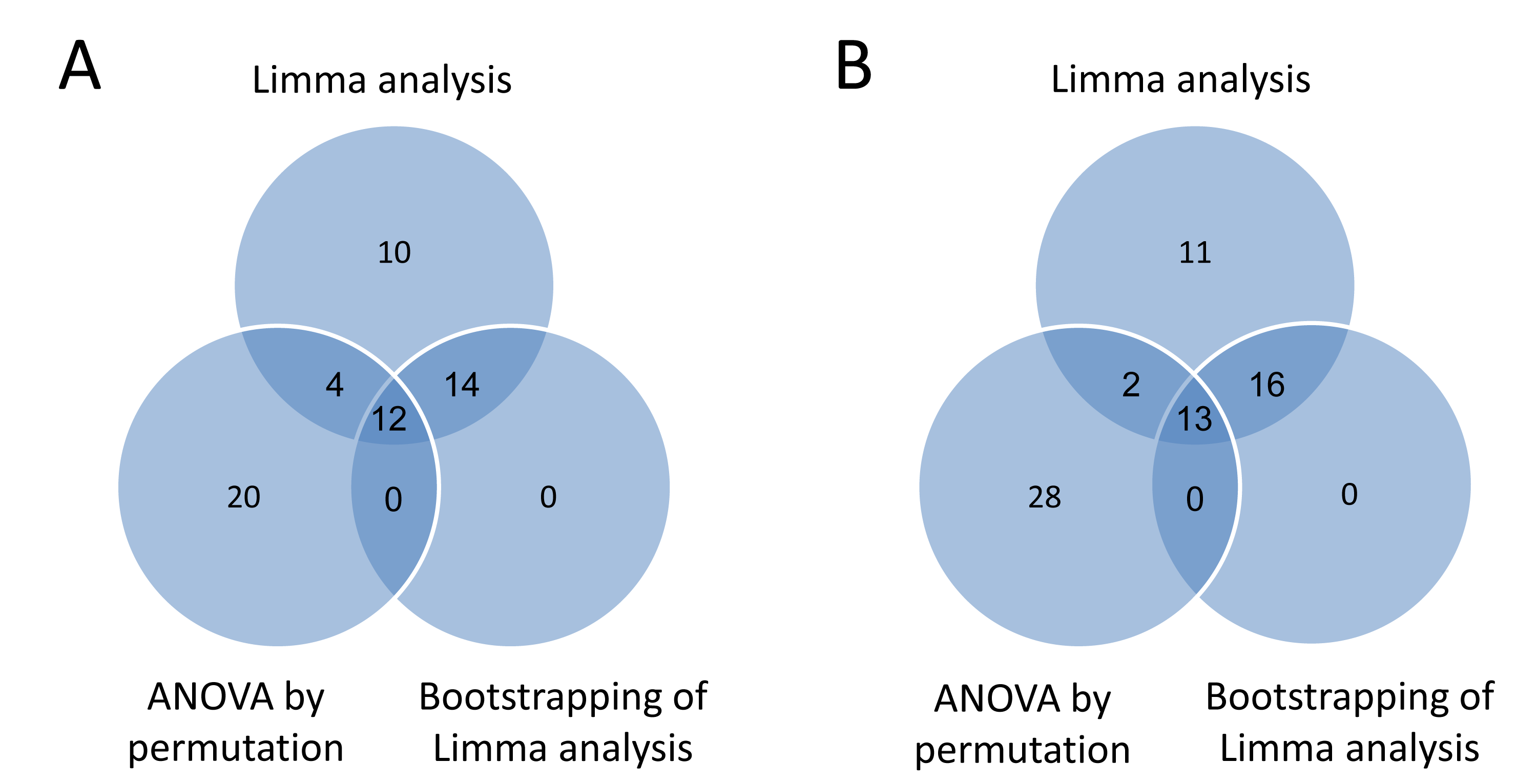

Supplement: Figure S2 — Venn diagrams depicting the relationships between three statistical procedures used to identify age-related differences in protein abundance. The three procedures were ANOVA with permutation, Limma and the Limma procedure applied to 1000 bootstrap samples of the data. The procedures were applied to DIGE protein expression profiles from aged Ae. aegypti females from two experimental cohorts. A. Cohort one (n = 773). B. Cohort two (n = 898). (TIF) [file pone.0058656.s002.tif]

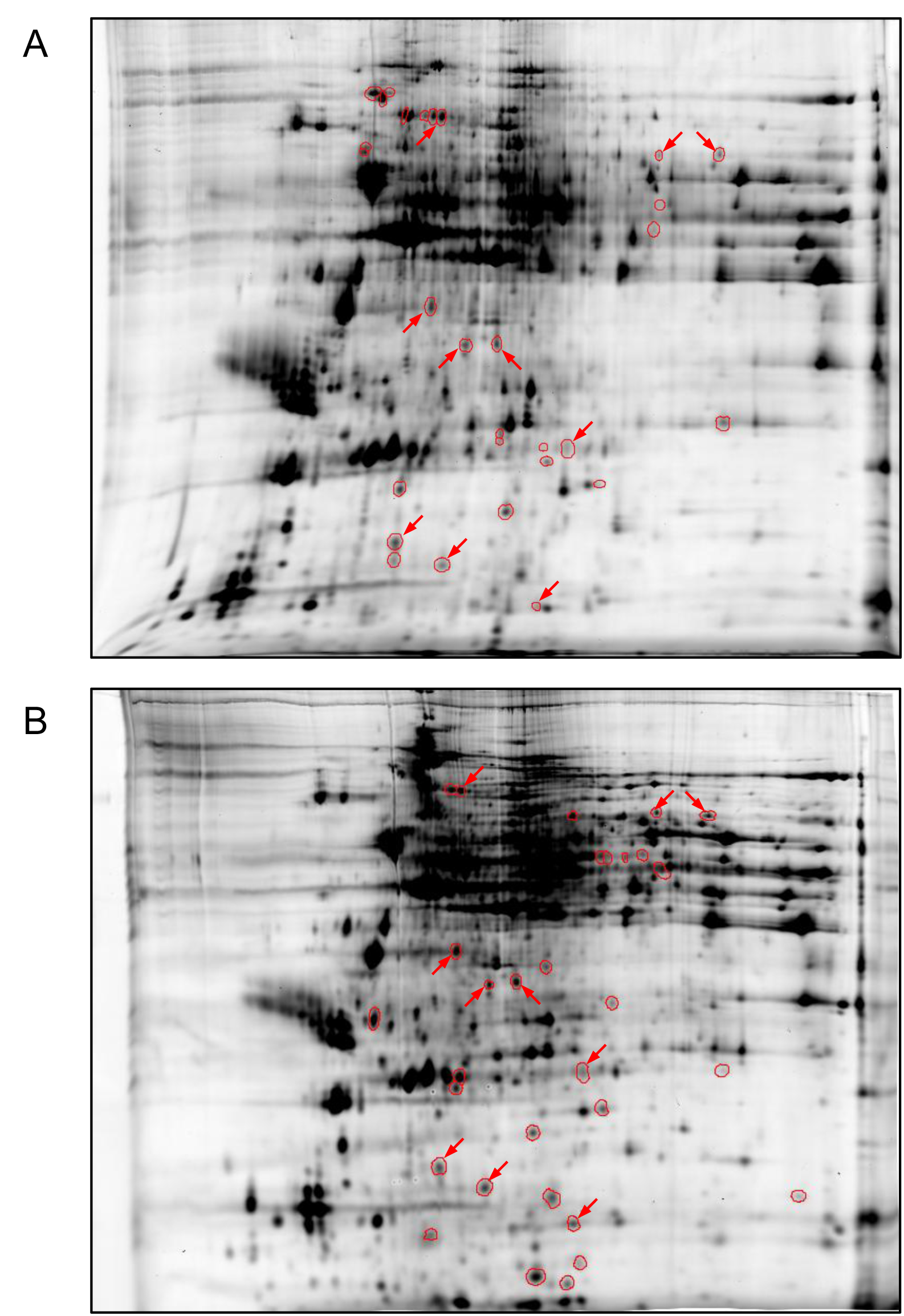

Supplement: Figure S3 — Age dependent spots detected from two Ae. aegypti cohorts using strict criteria for biomarker discovery. 2-D DIGE fusion images are shown for A. cohort one and B. cohort two. Circled spots indicate proteins determined to be significantly age-responsive in two out of three statistical analyses. Arrows indicate candidates shared between two mosquito cohorts. (TIF) [file pone.0058656.s003.tif]

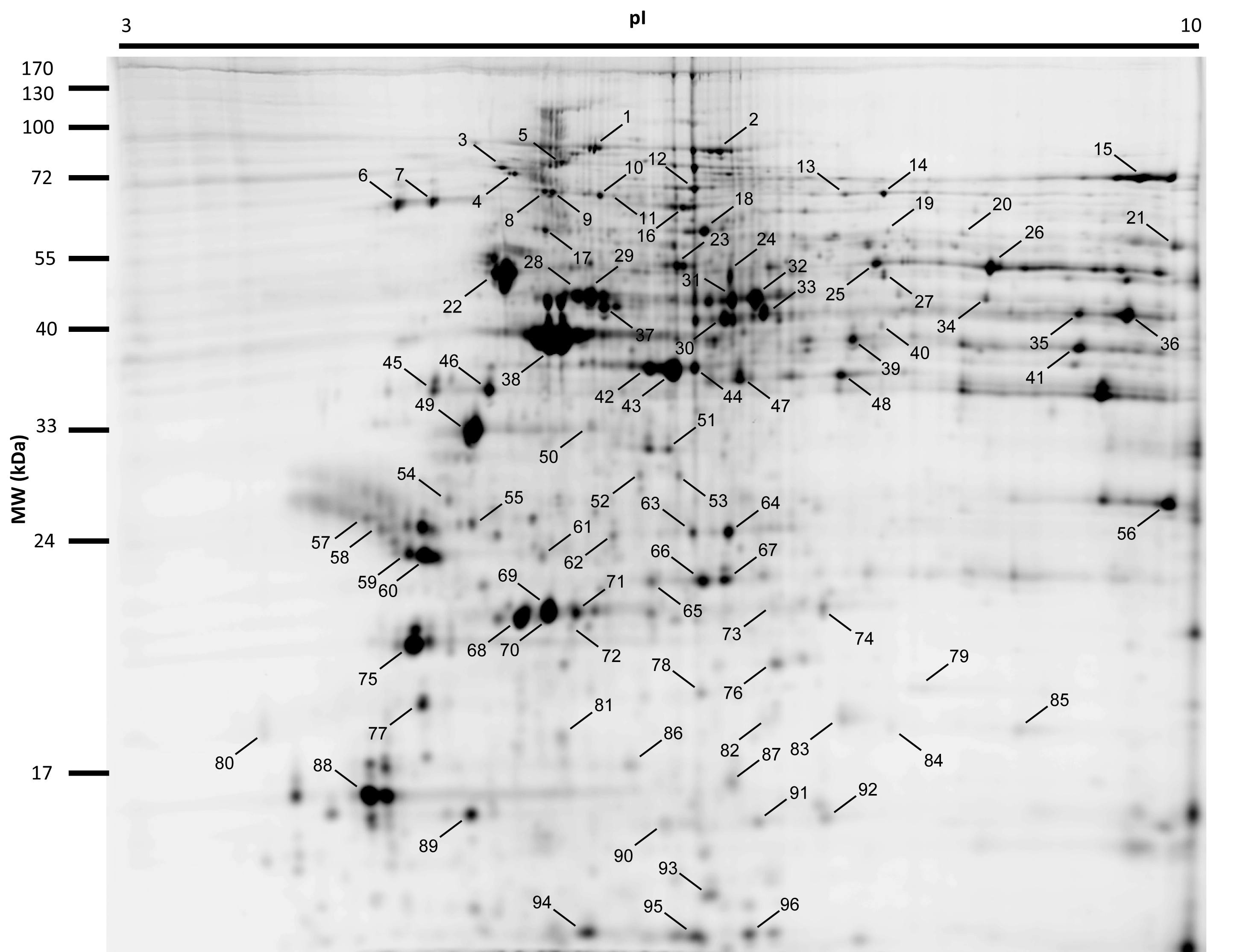

Supplement: Figure S4 — Two-dimensional gel electrophoresis map of the Ae. aegypti female head and thorax proteome showing spots identified by MALDI-TOF/TOF mass spectrometry. Numbers correspond to the protein identities in Table S3. (TIF) [file pone.0058656.s004.tif]

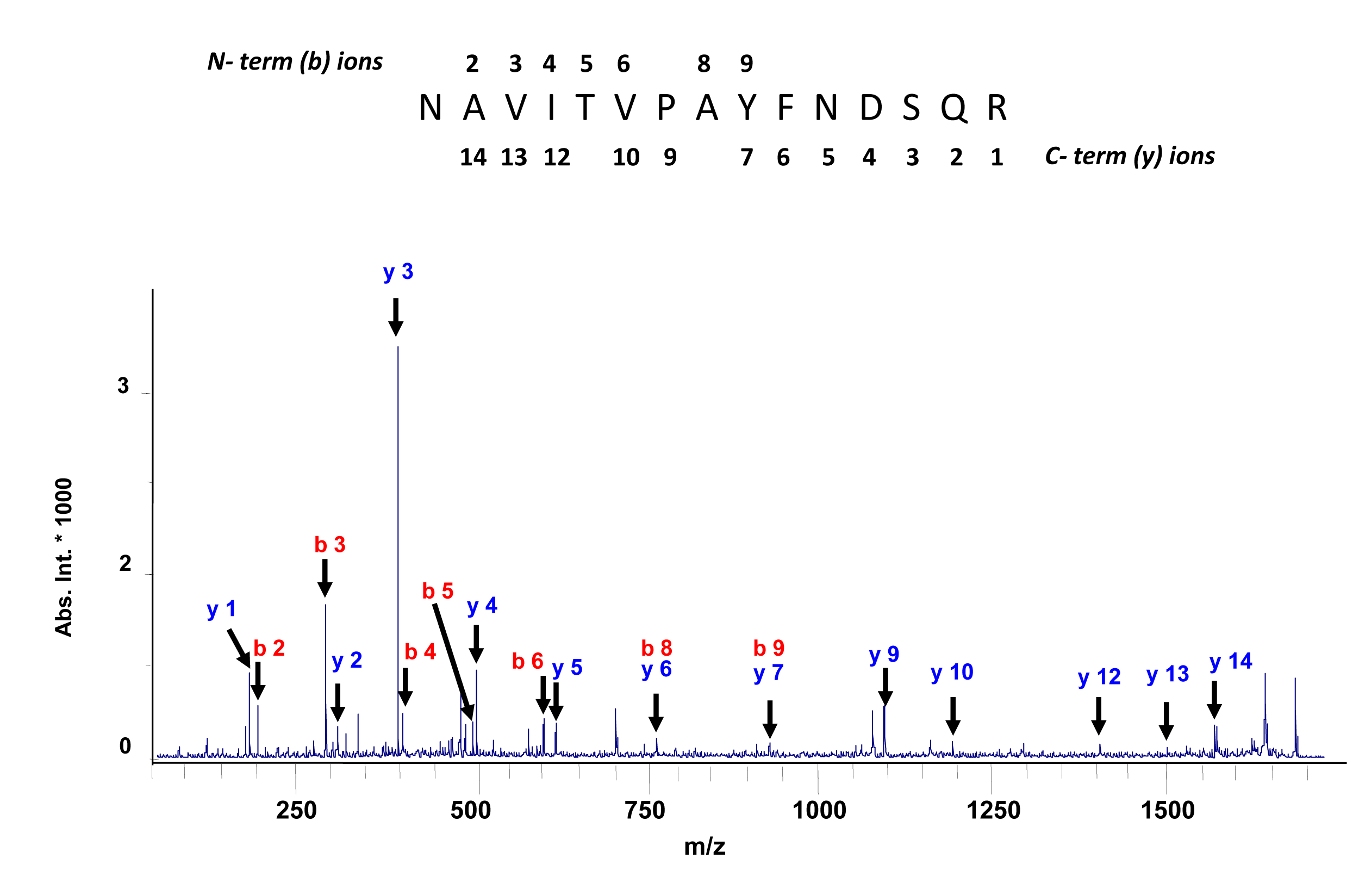

Supplement: Figure S5 — Annotated MS/MS spectra of a peptide from biomarker candidate HSP matching a conserved peptide from Ae. aegypti heat shock proteins. (TIF) [file pone.0058656.s005.tif]

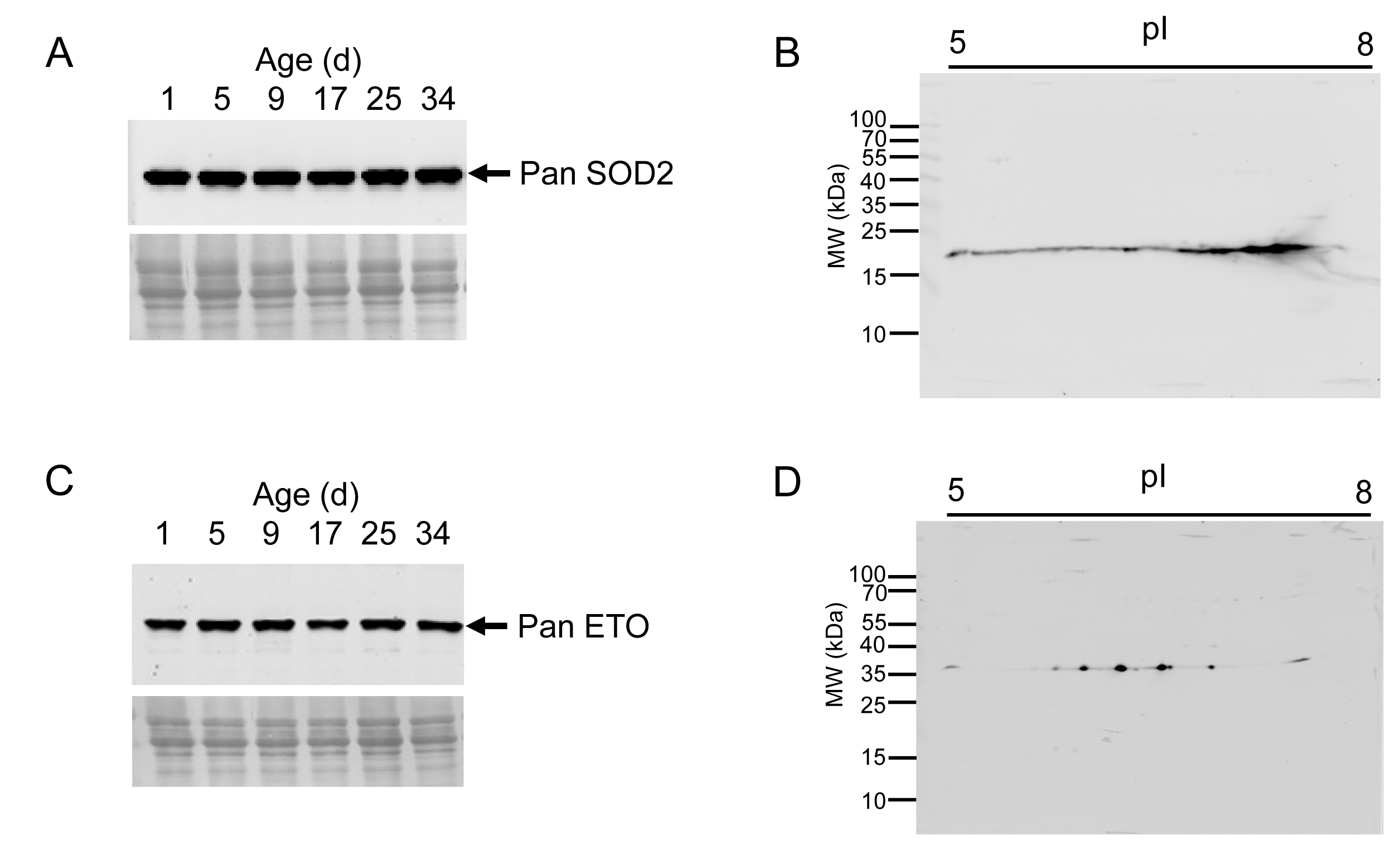

Supplement: Figure S6 — Western analyses of SOD2 and ETO proteins in aged Ae. aegypti mosquitoes. One dimensional western analysis of pools of five female Ae. aegypti using A. pan SOD2 and C. pan ETO antibodies demonstrated unchanging protein abundance from 1–34 d old. Below each panel is Direct Blue 71 stained total protein from each membrane indicating consistent sample loading. Two dimensional western analyses of one d old age samples using B. pan SOD2 and D. pan ETO polyclonal antibodies showed antibody recognition of potentially modified isoforms of the proteins, present as multiple protein spots or streaks on the horizontal (pI) axis. (TIF) [file pone.0058656.s006.tif]
