# Supplementary material for: Proteomic Biomarkers for Ageing the Mosquito Aedes aegypti to Determine Risk of Pathogen Transmission
Source: PLoS One. 2013 Mar 11;8(3):e58656. doi: 10.1371/journal.pone.0058656 (PMC3594161; doi:10.1371/journal.pone.0058656)
Supplement: Table S1 — Variance matrices for unrestricted and restricted mixed effects models fitted to cohort one for examining clustering and biological variance of protein expression profiles. (DOCX) [file pone.0058656.s007.docx]

|  |  |  | Heterogeneous model | | |  | Homogenous model | | |  | Log ratio test | | |
| --- | --- | --- | --- | --- | --- | --- | --- | --- | --- | --- | --- | --- | --- |
| Clusters fitted | Cluster | Age (d) | 1 | 17 | 34 |  | 1 | 17 | 34 |  | χ2 | df | P |
| 1 | 1 | 1 | 0.74 |  |  |  | 0.65 |  |  |  |  |  |  |
|  |  | 17 |  | 0.65 |  |  |  | 0.65 |  |  |  |  |  |
|  |  | 34 |  |  | 0.54 |  |  |  | 0.65 |  |  |  |  |
|  |  |  | Log likelihood = -10646.55 | | |  | Log likelihood = -10653.57 | | |  | 14.04 | 2 | 0.00089 |
| 5 | 1 | 1 | 0.18 |  |  |  | 0.09 |  |  |  |  |  |  |
|  |  | 17 |  | 0.04 |  |  |  | 0.09 |  |  |  |  |  |
|  |  | 34 |  |  | 0.02 |  |  |  | 0.09 |  |  |  |  |
|  | 2 | 1 | 0.86 |  |  |  | 0.14 |  |  |  |  |  |  |
|  |  | 17 |  | 0.17 |  |  |  | 0.14 |  |  |  |  |  |
|  |  | 34 |  |  | 0.11 |  |  |  | 0.14 |  |  |  |  |
|  | 3 | 1 | 2.43 |  |  |  | 2.54 |  |  |  |  |  |  |
|  |  | 17 |  | 2.98 |  |  |  | 2.54 |  |  |  |  |  |
|  |  | 34 |  |  | 2.77 |  |  |  | 2.54 |  |  |  |  |
|  | 4 | 1 | 0.72 |  |  |  | 0.30 |  |  |  |  |  |  |
|  |  | 17 |  | 0.15 |  |  |  | 0.30 |  |  |  |  |  |
|  |  | 34 |  |  | 0.08 |  |  |  | 0.30 |  |  |  |  |
|  | 5 | 1 | 0.25 |  |  |  | 0.20 |  |  |  |  |  |  |
|  |  | 17 |  | 0.05 |  |  |  | 0.20 |  |  |  |  |  |
|  |  | 34 |  |  | 0.02 |  |  |  | 0.20 |  |  |  |  |
|  |  |  | Log likelihood = -7415.13 | | |  | Log likelihood = -7595.18 | | |  | 360.1 | 8 | <0.01 |
