# Supplementary material for: Proteomic Biomarkers for Ageing the Mosquito Aedes aegypti to Determine Risk of Pathogen Transmission
Source: PLoS One. 2013 Mar 11;8(3):e58656. doi: 10.1371/journal.pone.0058656 (PMC3594161; doi:10.1371/journal.pone.0058656)
Supplement: Table S2 — Variance matrices for heterogeneous and homogenous mixed effects models fitted to cohort two for examining clustering and biological variance of protein expression profiles. (DOCX) [file pone.0058656.s008.docx]

|  |  |  | Heterogeneous model | | |  | Homogenous model | | |  | Log ratio test | | |
| --- | --- | --- | --- | --- | --- | --- | --- | --- | --- | --- | --- | --- | --- |
| Clusters fitted | Cluster | Age (d) | 1 | 17 | 34 |  | 1 | 17 | 34 |  | χ2 | df | P |
| 1 | 1 | 1 | 0.75 |  |  |  | 0.62 |  |  |  |  |  |  |
|  |  | 17 |  | 0.57 |  |  |  | 0.62 |  |  |  |  |  |
|  |  | 34 |  |  | 0.50 |  |  |  | 0.62 |  |  |  |  |
|  |  |  | Log likelihood = -12721.29 | | |  | Log likelihood = -12736.32 | | |  | 30.06 | 2 | 3E-07 |
|  |  |  |  |  |  |  |  |  |  |  |  |  |  |
| 5 | 1 | 1 | 0.11 |  |  |  | 0.06 |  |  |  |  |  |  |
|  |  | 17 |  | 0.04 |  |  |  | 0.06 |  |  |  |  |  |
|  |  | 34 |  |  | 0.03 |  |  |  | 0.06 |  |  |  |  |
|  | 2 | 1 | 0.34 |  |  |  | 0.11 |  |  |  |  |  |  |
|  |  | 17 |  | 0.08 |  |  |  | 0.11 |  |  |  |  |  |
|  |  | 34 |  |  | 0.06 |  |  |  | 0.11 |  |  |  |  |
|  | 3 | 1 | 1.39 |  |  |  | 1.21 |  |  |  |  |  |  |
|  |  | 17 |  | 1.06 |  |  |  | 1.21 |  |  |  |  |  |
|  |  | 34 |  |  | 0.93 |  |  |  | 1.21 |  |  |  |  |
|  | 4 | 1 | 4.62 |  |  |  | 4.61 |  |  |  |  |  |  |
|  |  | 17 |  | 5.13 |  |  |  | 4.61 |  |  |  |  |  |
|  |  | 34 |  |  | 4.77 |  |  |  | 4.61 |  |  |  |  |
|  | 5 | 1 | 0.61 |  |  |  | 0.36 |  |  |  |  |  |  |
|  |  | 17 |  | 0.04 |  |  |  | 0.36 |  |  |  |  |  |
|  |  | 34 |  |  | 0.05 |  |  |  | 0.36 |  |  |  |  |
|  |  |  | Log likelihood = -9226.665 | | |  | Log likelihood = -9356.896 | | |  | 260.5 | 8 | <0.01 |
