# Supplementary material for: Proteomic Biomarkers for Ageing the Mosquito Aedes aegypti to Determine Risk of Pathogen Transmission
Source: PLoS One. 2013 Mar 11;8(3):e58656. doi: 10.1371/journal.pone.0058656 (PMC3594161; doi:10.1371/journal.pone.0058656)
Supplement: Table S3 — Identities of proteins excised from preparative 2-D gels of female Ae . aegypti head and thorax tissue. Location of the spots is provided in Figure S4. Protein identities were determined using in-gel tryptic digestion and MALDI-TOF/TOF mass spectrometry as described in the text. Proteins matching at least two peptides with E-values <0.05 were accepted as confident identifications. ‡ An annotated MS/MS spectra of a peptide from spot 10 matching Ae. aegypti heat shock proteins is provided in Figure S5. (DOCX) [file pone.0058656.s009.docx]

| **Spot No.** | **Accession Number** | **Protein Name** | **pI** | **Mz** | **# MS/MS assigned** | **Sequence coverage (%)** | **Peptide sequence** | **Ion score** | **E-value** | **Delta Mass (ppm)** |
| --- | --- | --- | --- | --- | --- | --- | --- | --- | --- | --- |
| 1 | Q16RF4 | Paramyosin, long form | 5.48 | 103392 | 15 | 23 | R.RLHDYEVELR.R | 68 | 1.5e-005 | -10.76 |
|  |  |  |  |  |  |  | R.AQALSAEYGQFR.H | 67 | 1.7e-005 | -12.42 |
|  |  |  |  |  |  |  | K.QTSIEIEQLNAR.V | 59 | 0.00012 | -7.54 |
|  |  |  |  |  |  |  | K.RAQALSAEYGQFR.H + Deamidated (NQ) | 44 | 0.0036 | -5.18 |
|  |  |  |  |  |  |  | K.HTVELLHEEQER.I | 46 | 0.0024 | -9.79 |
|  |  |  |  |  |  |  | R.LRDIEVELEEER.R | 19 | 1.2 | 7.38 |
|  |  |  |  |  |  |  | R.QLQSTVDQYGIAQR.R + Deamidated (NQ) | 83 | 4.8e-007 | -7.11 |
|  |  |  |  |  |  |  | R.INDLTVSNASYASLR.T | 101 | 8.6e-009 | -11.57 |
|  |  |  |  |  |  |  | R.IQESEEHIESLQAR.L | 68 | 1.4e-005 | -9.98 |
|  |  |  |  |  |  |  | K.TIEKLEVQVTELNIR.I | 75 | 2.4e-006 | -8.58 |
|  |  |  |  |  |  |  | K.TRLEETITLYDQSQR.D | 64 | 4.3e-005 | -9.33 |
|  |  |  |  |  |  |  | R.QSAQLTEIQAHYEEIQR.Q + Deamidated (NQ) | 88 | 1.7e-007 | -6.79 |
|  |  |  |  |  |  |  | R.SILESTLHQVETELESVR.L | 88 | 1.7e-007 | -10.28 |
|  |  |  |  |  |  |  | R.TKLEQELQVLASDYEEVTR.E | 78 | 1.6e-006 | -13.74 |
|  |  |  |  |  |  |  | R.TFVTTSTVPGSQVYLVQETTR.T | 123 | 4.7e-011 | -9.26 |
| 2 | Q175A3 | 2-oxoglutarate dehydrogenase | 6.69 | 119512 | 13 | 16 | R.FDTAINSTR.D | 35 | 0.02 | -17.29 |
|  |  |  |  |  |  |  | K.TRAEQFYR.G | 26 | 0.14 | -18.38 |
|  |  |  |  |  |  |  | K.YHLGTYIER.L | 47 | 0.0016 | -13.01 |
|  |  |  |  |  |  |  | K.DVIIDLVSYR.R | 58 | 0.00013 | -8.08 |
|  |  |  |  |  |  |  | K.NQGYWTYIEPR.F | 57 | 0.00017 | -10.99 |
|  |  |  |  |  |  |  | R.LEQISPFPYDLIK.A | 56 | 0.00025 | -6.49 |
|  |  |  |  |  |  |  | R.DPASVHASWDAYFR.N | 4 | 24 | -47.99 |
|  |  |  |  |  |  |  | R.NNSYEAPPSLAPIPR.N | 111 | 7.3e-010 | -9.18 |
|  |  |  |  |  |  |  | R.SSFSEMTDGTEFQR.I + Oxidation (M) | 38 | 0.008 | -9.20 |
|  |  |  |  |  |  |  | K.VAPTGVIEETLVHIGNR.F | 84 | 3.6e-007 | -8.05 |
|  |  |  |  |  |  |  | R.FETPNIMNYTNEEKR.L + Oxidation (M) | 61 | 7.1e-005 | -10.41 |
|  |  |  |  |  |  |  | R.IDDKLIDDHLAVQAIIR.S | 140 | 9.2e-013 | -7.37 |
|  |  |  |  |  |  |  | R.NHVPASQYLGSAVPALAGGSSAVGTR.I + Deamidated (NQ) | 89 | 1.3e-007 | -6.50 |
|  | Q175A4 | 2-oxoglutarate dehydrogenase | 6.42 | 115048 | 13 | 17 | Matches same set of peptides | | | |
| 3 | Q16FA5 | Heat shock protein | 4.94 | 81825 | 8 | 14 | K.TKPIWTR.N | 43 | 0.003 | 120 |
|  |  |  |  |  |  |  | K.FYDQFSK.N | 39 | 0.01 | 120 |
|  |  |  |  |  |  |  | K.HFSVEGQLDFR.A | 72 | 5.5e-006 | 91.3 |
|  |  |  |  |  |  |  | K.GVVDSEDLPLNISR.E | 40 | 0.0082 | 86.6 |
|  |  |  |  |  |  |  | K.SLTNDWEDHLAVK.H | 22 | 0.54 | 106 |
|  |  |  |  |  |  |  | R.NADDISQEEYGEFYK.S | 56 | 0.00025 | 99.4 |
|  |  |  |  |  |  |  | R.FNTSASGDEYCSLGDYVGR.M | 158 | 1.8e-014 | 97.5 |
|  |  |  |  |  |  |  | K.SNDDEQYVWESSAGGSFTVR.Q | 135 | 3.4e-012 | 101 |
| 4 | Q1HR69 | Heat shock cognate 70 | 5.06 | 72356 | 11 | 19 | K.IVITNDQNR.L | 44 | 0.0026 | 120 |
|  |  |  |  |  |  |  | K.FDLTGIPPAPR.G | 90 | 5.2e-008 | 119 |
|  |  |  |  |  |  |  | K.EFFNGKEPSR.G + Deamidated (NQ) | 66 | 2.2e-005 | 128 |
|  |  |  |  |  |  |  | K.DVDEIVLVGGSTR.I | 108 | 1.6e-009 | 106 |
|  |  |  |  |  |  |  | R.AKFEELNMDLFR.S | 71 | 9.1e-006 | 107 |
|  |  |  |  |  |  |  | R.ITPSYVAFTADGER.L | 87 | 1.9e-007 | 120 |
|  |  |  |  |  |  |  | K.NQLTTNPENTVFDAKR.L | 83 | 6.3e-007 | 104 |
|  |  |  |  |  |  |  | K.VTHAVVTVPAYFNDAQR.Q | 126 | 2.5e-011 | 113 |
|  |  |  |  |  |  |  | K.DNHLLGKFDLTGIPPAPR.G | 56 | 0.00018 | 112 |
|  |  |  |  |  |  |  | K.KVTHAVVTVPAYFNDAQR.Q | 82 | 6.1e-007 | 106 |
|  |  |  |  |  |  |  | R.IEIESFYEGDDFSETLTR.A | 97 | 2.7e-008 | 114 |
| 5 | Q16SH1 | Spermatogenesis associated factor | 5.30 | 89434 | 8 | 10 | K.MDELQLFR.G | 57 | 0.00013 | 121 |
|  |  |  |  |  |  |  | R.QAIEAEIRR.E | 23 | 0.24 | 107 |
|  |  |  |  |  |  |  | R.GVLFYGPPGCGK.T | 45 | 0.0027 | 99.4 |
|  |  |  |  |  |  |  | R.EIDIGIPDATGR.L | 27 | 0.15 | 94.9 |
|  |  |  |  |  |  |  | K.YEMFAQTLQQSR.G | 13 | 5.4 | 83.9 |
|  |  |  |  |  |  |  | K.AIANECQANFISVK.G | 39 | 0.011 | 85.6 |
|  |  |  |  |  |  |  | R.KYEMFAQTLQQSR.G | 88 | 1.7e-007 | 79.7 |
|  |  |  |  |  |  |  | K.VTQGFSGADLTEICQR.A | 134 | 4.1e-012 | 98.9 |
| 6 | Q17H75 | Tropomyosin invertebrate | 4.71 | 32759 | 5 | 21 | R.ALLCEQQAR.D | 29 | 0.11 | 101 |
|  |  |  |  |  |  |  | K.IVELEEELR.V | 33 | 0.033 | 104 |
|  |  |  |  |  |  |  | K.ALQNAESEVAALNR.R | 24 | 0.31 | 79.7 |
|  |  |  |  |  |  |  | R.IQLLEEDLERSEER.L | 51 | 0.00069 | 90.2 |
|  |  |  |  |  |  |  | R.FMAEEADKKYDEVAR.K | 68 | 1.4e-005 | 96.1 |
| 7 | Q17H75 | Tropomyosin invertebrate | 4.71 | 32759 | 5 | 21 | R.ALLCEQQAR.D | 18 | 1.3 | 123 |
|  |  |  |  |  |  |  | K.IVELEEELR.V | 31 | 0.05 | 117 |
|  |  |  |  |  |  |  | K.ALQNAESEVAALNRR.I | 14 | 2.5 | 99.7 |
|  |  |  |  |  |  |  | R.IQLLEEDLERSEER.L | 37 | 0.019 | 99.9 |
|  |  |  |  |  |  |  | R.FMAEEADKKYDEVAR.K | 54 | 0.00033 | 103 |
| 8 | Q1HQZ5 | Heat shock cognate 70 | 5.31 | 71388 | 7 | 13 | K.FELSGIPPAPR.G | 63 | 3.7e-005 | 53.5 |
|  |  |  |  |  |  |  | R.LVNHFAQEFKR.K | 47 | 0.0017 | 55.2 |
|  |  |  |  |  |  |  | K.ASIHDIVLVGGSTR.I | 30 | 0.072 | 39.0 |
|  |  |  |  |  |  |  | R.TTPSYVAFTDTER.L | 37 | 0.021 | 65.2 |
|  |  |  |  |  |  |  | K.STAGDTHLGGEDFDNR.L | 93 | 4.7e-008 | 64.6 |
|  |  |  |  |  |  |  | K.DNNLLGKFELSGIPPAPR.G | 122 | 5.4e-011 | 60.8 |
|  |  |  |  |  |  |  | K.TVSNAVVTVPAYFNDSQR.Q | 37 | 0.021 | 61.9 |
| 9 | Q1HQZ5 | Heat shock cognate 70 | 5.31 | 71388 | 12 | 22 | K.FELSGIPPAPR.G | 77 | 9.4e-007 | 98.1 |
|  |  |  |  |  |  |  | K.DAGTISGLNVLR.I | 59 | 9.6e-005 | 90.7 |
|  |  |  |  |  |  |  | R.FEELNADLFR.S | 34 | 0.031 | 105 |
|  |  |  |  |  |  |  | R.LVNHFAQEFKR.K | 70 | 7.9e-006 | 76.4 |
|  |  |  |  |  |  |  | K.ASIHDIVLVGGSTR.I | 61 | 3.4e-005 | 100 |
|  |  |  |  |  |  |  | R.ARFEELNADLFR.S | 64 | 2.9e-005 | 113 |
|  |  |  |  |  |  |  | R.TTPSYVAFTDTER.L | 45 | 0.0031 | 99.9 |
|  |  |  |  |  |  |  | K.STAGDTHLGGEDFDNR.L | 103 | 4.9e-009 | 116 |
|  |  |  |  |  |  |  | K.NQVAMNPTNTIFDAKR.L | 57 | 0.00019 | 98.6 |
|  |  |  |  |  |  |  | K.DNNLLGKFELSGIPPAPR.G | 139 | 6.8e-013 | 95.9 |
|  |  |  |  |  |  |  | K.TVSNAVVTVPAYFNDSQR.Q | 98 | 1.4e-008 | 98.3 |
|  |  |  |  |  |  |  | K.WLDANQLAEKEEYEHR.Q | 122 | 6.1e-011 | 114 |
| 10 | B7TBH7 | Heat shock 70 Cb (HSP) | 5.62 | 70557 | 1 | 2 | K.NAVITVPAYFNDSQR.Q‡ | 59 | 0.00012 | -0.33 |
|  | B7TBH2 | Heat shock 70 Aa | 5.80 | 70751 | 1 | 2 | K.NAVITVPAYFNDSQR.Q | 59 | 0.00012 | -0.33 |
|  | B7TBH3 | Heat shock 70 Ab | 5.90 | 70744 | 1 | 2 | K.NAVITVPAYFNDSQR.Q | 59 | 0.00012 | -0.33 |
|  | B7TBH4 | Heat shock 70 Ba | 5.80 | 70720 | 1 | 2 | K.NAVITVPAYFNDSQR.Q | 59 | 0.00012 | -0.33 |
|  | B7TBH5 | Heat shock 70 Bb | 5.80 | 70575 | 1 | 2 | K.NAVITVPAYFNDSQR.Q | 59 | 0.00012 | -0.33 |
|  | B7TBH6 | Heat shock 70 Ca | 5.70 | 70489 | 1 | 2 | K.NAVITVPAYFNDSQR.Q | 59 | 0.00012 | -0.33 |
| 11 | B7TBH2 | Heat shock 70 Aa | 5.80 | 70751 | 1 | 2 | K.NAVITVPAYFNDSQR.Q | 139 | 5e-013 | 127 |
|  | B7TBH3 | Heat shock 70 Ab | 5.90 | 70744 | 1 | 2 | K.NAVITVPAYFNDSQR.Q | 139 | 5e-013 | 127 |
|  | B7TBH4 | Heat shock 70 Ba | 5.80 | 70720 | 1 | 2 | K.NAVITVPAYFNDSQR.Q | 139 | 5e-013 | 127 |
|  | B7TBH5 | Heat shock 70 Bb | 5.80 | 70575 | 1 | 2 | K.NAVITVPAYFNDSQR.Q | 139 | 5e-013 | 127 |
|  | B7TBH6 | Heat shock 70 Ca | 5.70 | 70489 | 1 | 2 | K.NAVITVPAYFNDSQR.Q | 139 | 5e-013 | 127 |
|  | B7TBH7 | Heat shock 70 Cb | 5.62 | 70557 | 1 | 2 | K.NAVITVPAYFNDSQR.Q | 139 | 5e-013 | 127 |
| 12 | Q16LR5 | NADH-ubiquinone oxidoreductase | 6.54 | 80119 | 14 | 27 | R.FCYHER.L | 15 | 1.3 | -25.48 |
|  |  |  |  |  |  |  | R.FACDGLKR.Q | 11 | 5.6 | -25.64 |
|  |  |  |  |  |  |  | R.YEAPLLNTR.L | 45 | 0.003 | -20.53 |
|  |  |  |  |  |  |  | K.QATYVNTEGR.A | 32 | 0.053 | -25.98 |
|  |  |  |  |  |  |  | K.KPLIIVGANQLAR.K | 89 | 9.3e-008 | -18.58 |
|  |  |  |  |  |  |  | K.VLFLLGADAGAIKK.E | 34 | 0.032 | -21.89 |
|  |  |  |  |  |  |  | R.AQQTLVAVTPPGLAR.E | 48 | 0.0014 | -20.05 |
|  |  |  |  |  |  |  | K.QLADFFMTDPITR.A + Oxidation (M) | 28 | 0.15 | -17.19 |
|  |  |  |  |  |  |  | R.FASEVAGVDDLGTTGR.G | 96 | 2.4e-008 | -13.69 |
|  |  |  |  |  |  |  | R.ENEEINEEWLSDKSR.F | 33 | 0.054 | -15.29 |
|  |  |  |  |  |  |  | R.ALSEIAGAPLPYDTLDELR.S | 75 | 3.5e-006 | -18.94 |
|  |  |  |  |  |  |  | K.VNLSYEYEHLGNDPSLVR.D | 59 | 0.00014 | -12.98 |
|  |  |  |  |  |  |  | R.LGSETLCTEQKFPTDGSGTDFR.S | 96 | 2.5e-008 | -14.43 |
|  |  |  |  |  |  |  | R.NPSGELEAVEWESALITIAQALR.G | 52 | 0.00062 | -18.15 |
| 13 | Q17E81 | Glycerol-3-phosphate dehydrogenase | 7.86 | 81660 | 8 | 13 | K.AYDFVAGDR.N | 40 | 0.0067 | -1.16 |
|  |  |  |  |  |  |  | K.HLAISYGDR.A | 21 | 0.76 | -1.42 |
|  |  |  |  |  |  |  | K.GYVSIPDIKR.A | 16 | 2.4 | -23.28 |
|  |  |  |  |  |  |  | K.AILGLDIEQYR.M | 44 | 0.004 | -9.19 |
|  |  |  |  |  |  |  | K.CVINATGPFTDSIR.K | 41 | 0.0091 | -11.08 |
|  |  |  |  |  |  |  | R.GAIVYYDGQQDDAR.M | 74 | 4e-006 | -9.95 |
|  |  |  |  |  |  |  | K.LHPEFPYIDAEVR.Y | 35 | 0.033 | -11.15 |
|  |  |  |  |  |  |  | R.TYGDAEVSGEELHDILR.E | 40 | 0.011 | -8.21 |
| 14 | Q17E81 | Glycerol-3-phosphate dehydrogenase | 7.86 | 81660 | 12 | 20 | K.AYDFVAGDR.N | 35 | 0.021 | -9.71 |
|  |  |  |  |  |  |  | K.HLAISYGDR.A | 23 | 0.53 | -13.29 |
|  |  |  |  |  |  |  | K.GYVSIPDIKR.A | 30 | 0.087 | -21.84 |
|  |  |  |  |  |  |  | K.AILGLDIEQYR.M | 69 | 1.4e-005 | -17.12 |
|  |  |  |  |  |  |  | R.EDALELFPMLR.G | 27 | 0.23 | -26.39 |
|  |  |  |  |  |  |  | R.EYACTCIDMVAR.R | 30 | 0.087 | -28.84 |
|  |  |  |  |  |  |  | K.CVINATGPFTDSIR.K | 52 | 0.0007 | -18.16 |
|  |  |  |  |  |  |  | R.GAIVYYDGQQDDAR.M | 77 | 1.8e-006 | -14.46 |
|  |  |  |  |  |  |  | K.LHPEFPYIDAEVR.Y | 73 | 5.4e-006 | -16.07 |
|  |  |  |  |  |  |  | R.TYGDAEVSGEELHDILR.E | 89 | 1.2e-007 | -18.85 |
|  |  |  |  |  |  |  | R.FAVVAEQEEIRKEQER.L | 44 | 0.0043 | -17.46 |
|  |  |  |  |  |  |  | K.ACEHFLHTQMGQQVNR.Q + Oxidation (M) | 26 | 0.23 | -16.79 |
| 15 | Q16KR4 | Aconitase, mitochondrial | 8.67 | 86411 | 7 | 9 | K.IPFNVTPGSEQIR.A | 82 | 6e-007 | -0.71 |
|  |  |  |  |  |  |  | K.NQVTGEWAGVPDVAR.F | 76 | 2.5e-006 | -2.78 |
|  |  |  |  |  |  |  | R.WVAVGDENYGEGSSR.E | 101 | 7.1e-009 | 0.97 |
|  |  |  |  |  |  |  | K.SKIPFNVTPGSEQIR.A | 31 | 0.087 | -1.11 |
|  |  |  |  |  |  |  | K.LNHSFNEQQIAWFK.A | 100 | 1e-008 | 5.84 |
|  |  |  |  |  |  |  | K.IKNQVTGEWAGVPDVAR.F | 57 | 0.00018 | -1.87 |
|  |  |  |  |  |  |  | K.ILYGHLDDPANQDIQR.G | 117 | 1.7e-010 | 2.57 |
| 16 | Q16SE4 | Succinate dehydrogenase | 6.35 | 72807 | 14 | 31 | K.KPVEEHWR.K | 61 | 7.4e-005 | -18.65 |
|  |  |  |  |  |  |  | K.NTVLATGGYGR.A | 18 | 1.4 | -12.02 |
|  |  |  |  |  |  |  | R.SKNTVLATGGYGR.A | 48 | 0.0015 | -20.65 |
|  |  |  |  |  |  |  | R.GEGGYLINSQGER.F | 62 | 6.3e-005 | -21.50 |
|  |  |  |  |  |  |  | R.LGANSLLDLVVFGR.A | 123 | 4.5e-011 | -19.42 |
|  |  |  |  |  |  |  | K.TMQTHAAVFREEK.T | 44 | 0.0041 | -19.89 |
|  |  |  |  |  |  |  | K.AVIELENYGMPFSR.T | 81 | 6.7e-007 | -18.73 |
|  |  |  |  |  |  |  | K.GSDWLGDQDAIHYMTR.E | 130 | 1e-011 | -17.91 |
|  |  |  |  |  |  |  | R.LPGISETAMIFAGVDVTR.E | 61 | 7.9e-005 | -17.07 |
|  |  |  |  |  |  |  | K.HRVDEYDYSKPLEGQQK.K | 49 | 0.0014 | -19.31 |
|  |  |  |  |  |  |  | K.IADIKPNAGEASVANLDWVR.N | 169 | 1.2e-015 | -18.76 |
|  |  |  |  |  |  |  | K.DHVYLQLHHLPPEQLAQR.L | 72 | 6.7e-006 | -20.19 |
|  |  |  |  |  |  |  | K.EYPVVDHTYDAVVVGAGGAGLR.A | 171 | 8.4e-016 | -16.18 |
|  |  |  |  |  |  |  | R.AYFSCTSAHTCTGDGTAMVAR.A | 123 | 4.4e-011 | -17.98 |
| 17 | Q16PM9 | Chaperonin-60kD, ch60 | 5.47 | 61155 | 9 | 24 | K.GANPVEIRR.G | 48 | 0.00049 | 111 |
|  |  |  |  |  |  |  | R.AIGDLISEAMKR.V | 33 | 0.043 | 82.8 |
|  |  |  |  |  |  |  | R.NVILEQSWGSPK.I | 82 | 4.6e-007 | 92.3 |
|  |  |  |  |  |  |  | R.GYISPYFINSSK.G | 57 | 0.00016 | 98.2 |
|  |  |  |  |  |  |  | R.AAVEEGIVPGGGTALLR.C | 143 | 1.9e-013 | 107 |
|  |  |  |  |  |  |  | K.ISSVQSIIPALELANSAR.K | 78 | 7.5e-007 | 92.8 |
|  |  |  |  |  |  |  | R.AVTSPEEIAQVATISANGDR.A + Deamidated (NQ) | 89 | 1e-007 | 121 |
|  |  |  |  |  |  |  | R.VEQIRDQIAETTSEYEKEK.L | 66 | 2.2e-005 | 106 |
|  |  |  |  |  |  |  | R.KPLVIIAEDVDGEALSTLVVNR.L | 136 | 5.4e-013 | 112 |
| 18 | Q17M99 | Malic enzyme | 7.22 | 72552 | 12 | 22 | R.AIVVTDGER.I | 18 | 1.6 | -46.36 |
|  |  |  |  |  |  |  | R.GYVYEVLR.N | 47 | 0.0013 | -28.88 |
|  |  |  |  |  |  |  | K.GLAFTLEER.Q | 27 | 0.16 | -22.96 |
|  |  |  |  |  |  |  | R.NWPEHDVR.A | 46 | 0.0018 | -32.84 |
|  |  |  |  |  |  |  | K.WPPQQEAPK.R | 17 | 1.6 | -22.57 |
|  |  |  |  |  |  |  | R.QTLGIHGLQPAR.F + Deamidated (NQ) | 47 | 0.0019 | -125.06 |
|  |  |  |  |  |  |  | K.AMQAEGCGLQEAR.D | 23 | 0.45 | -17.49 |
|  |  |  |  |  |  |  | R.FKSQEEQLELCR.I | 61 | 7.6e-005 | -23.28 |
|  |  |  |  |  |  |  | K.YLYLVDLQDRNEK.L | 42 | 0.0063 | -19.82 |
|  |  |  |  |  |  |  | K.NNARPIIFALSNPTSK.A | 79 | 1.3e-006 | -20.31 |
|  |  |  |  |  |  |  | R.VSGKEYDDFIDEFMEAVVKR.Y | 107 | 2.1e-009 | -14.22 |
|  |  |  |  |  |  |  | K.RYGQNTLIQFEDFGNHNAFR.F | 37 | 0.02 | -16.65 |
| 19 | Q16F38 | Pyruvate kinase (PK) | 7.10 | 58048 | 8 | 19 | R.CPIIAVTR.F | 25 | 0.3 | -27.58 |
|  |  |  |  |  |  |  | K.EAEAALWHR.N | 20 | 0.77 | -13.96 |
|  |  |  |  |  |  |  | R.VQYGMDFGKER.G | 24 | 0.34 | -19.43 |
|  |  |  |  |  |  |  | R.GFLKPGNPVVVVTGWK.Q | 45 | 0.0034 | -21.53 |
|  |  |  |  |  |  |  | R.LNFSHGSHEYHANTIK.N | 45 | 0.0034 | -21.53 |
|  |  |  |  |  |  |  | K.QGKPFPLAIALDTKGPEIR.T | 44 | 0.0043 | -21.94 |
|  |  |  |  |  |  |  | K.KQGKPFPLAIALDTKGPEIR.T | 28 | 0.18 | -21.61 |
|  |  |  |  |  |  |  | K.DKSDLQFGVEQGVDVIFASFIR.N | 15 | 2.9 | -31.34 |
|  | Q16LP4 | Pyruvate kinase | 7.10 | 56080 | 8 | 19 | Matches same set of peptides | | | |
|  | Q16LP5 | Pyruvate kinase | 7.55 | 56752 | 8 | 19 | Matches same set of peptides | | | |
| 20 | Q16RF4 | Paramyosin, long form (Pm) | 5.48 | 103392 | 11 | 17 | R.RIIGKLEAR.L | 19 | 0.74 | -23.75 |
|  |  |  |  |  |  |  | R.ADVAESNLHLVR.A | 37 | 0.019 | -17.75 |
|  |  |  |  |  |  |  | R.QVEVQLEEATSR.I | 35 | 0.032 | -14.25 |
|  |  |  |  |  |  |  | K.QTSIEIEQLNAR.V | 74 | 3.6e-006 | -12.39 |
|  |  |  |  |  |  |  | K.HTVELLHEEQER.I | 66 | 3e-005 | -19.44 |
|  |  |  |  |  |  |  | R.QLQSTVDQYGIAQR.R | 89 | 1.2e-007 | -11.37 |
|  |  |  |  |  |  |  | R.INDLTVSNASYASLR.T | 90 | 1e-007 | -9.64 |
|  |  |  |  |  |  |  | R.QLQSTVDQYGIAQRR.I | 17 | 2 | 0.17 |
|  |  |  |  |  |  |  | R.QSAQLTEIQAHYEEIQR.Q | 92 | 7.1e-008 | -14.32 |
|  |  |  |  |  |  |  | R.ELEAAEDRADVAESNLHLVR.A | 81 | 7.3e-007 | -14.95 |
|  |  |  |  |  |  |  | R.TKLEQELQVLASDYEEVTR.E | 51 | 0.00074 | -12.76 |
|  |  |  |  |  |  |  | R.TFVTTSTVPGSQVYLVQETTR.T | 84 | 3.7e-007 | -12.28 |
| 21 | Q17A27 | Pyrroline-5-carboxylate dehydrogenase | 8.9 | 63634 | 14 | 32 | K.FYWADKK.L | 40 | 0.0077 | 124 |
|  |  |  |  |  |  |  | K.AGGPHYVLR.W | 73 | 1.8e-006 | 120 |
|  |  |  |  |  |  |  | K.AADLMAGPYR.A | 56 | 0.00021 | 113 |
|  |  |  |  |  |  |  | R.SSFEFCGQK.C | 63 | 4.9e-005 | 116 |
|  |  |  |  |  |  |  | K.WDRTPISER.I | 29 | 0.11 | 121 |
|  |  |  |  |  |  |  | K.TATETQVKWDR.T | 49 | 0.0012 | 97.7 |
|  |  |  |  |  |  |  | R.MYVPESLWPQVK.E + Oxidation (M) | 67 | 1.7e-005 | 115 |
|  |  |  |  |  |  |  | R.QVGENINIYNNFPR.L | 126 | 2.2e-011 | 107 |
|  |  |  |  |  |  |  | K.GYFIEPTIVQSNDPK.D | 42 | 0.0044 | 116 |
|  |  |  |  |  |  |  | R.FALTGAVFSKDEAFLKR.A | 73 | 2e-006 | 111 |
|  |  |  |  |  |  |  | K.TASTLEDVPIIIGSEEFR.T | 152 | 3e-014 | 129 |
|  |  |  |  |  |  |  | K.TVIQAEIDSAAELIDFIR.M | 65 | 1.6e-005 | 113 |
|  |  |  |  |  |  |  | K.ETFVPLNEVDYQYMRE.- + Oxidation (M) | 26 | 0.26 | 132 |
|  |  |  |  |  |  |  | K.IGDVTDFSTFTSAVIDDKAFNR.I | 123 | 3.2e-011 | 136 |
| 22 | Q17FL3 | ATP synthase subunit beta | 5.02 | 53940 | 11 | 35 | R.IINVIGEPIDER.G | 68 | 1.1e-005 | 63.3 |
|  |  |  |  |  |  |  | K.AHGGYSVFAGVGER.T | 106 | 2.7e-009 | 62.8 |
|  |  |  |  |  |  |  | R.VALTGLTVAEYFR.D | 67 | 1.3e-005 | 68.5 |
|  |  |  |  |  |  |  | K.VALVYGQMNEPPGAR.A | 81 | 6.6e-007 | 70.3 |
|  |  |  |  |  |  |  | R.LVLEVAQHLGENTVR.T | 118 | 1.1e-010 | 76.9 |
|  |  |  |  |  |  |  | R.IMDPNIIGAEHYNIAR.G | 104 | 3.4e-009 | 75.0 |
|  |  |  |  |  |  |  | R.DQEGQDVLLFIDNIFR.F | 116 | 2.7e-010 | 80.0 |
|  |  |  |  |  |  |  | R.VLDTGSPIRIPVGAETLGR.I | 35 | 0.011 | 99.6 |
|  |  |  |  |  |  |  | R.FLSQPFQVAEVFTGHAGK.L | 27 | 0.17 | 90.9 |
|  |  |  |  |  |  |  | R.AIAELGIYPAVDPLDSTSR.I | 72 | 4.3e-006 | 102 |
|  |  |  |  |  |  |  | R.IPSAVGYQPTLATDMGSMQER.I | 93 | 5.7e-008 | 106 |
|  | Q17H12 | ATP synthase subunit beta | 5.03 | 53878 | 11 | 35 | Matches same set of peptides | | | |
|  | Q1HR61 | ATP synthase subunit beta | 5.03 | 53937 | 11 | 35 | Matches same set of peptides | | | |
| 23 | Q174D6 | Dihydrolipoyl dehydrogenase | 6.36 | 54139 | 8 | 25 | K.FPFMANSR.A + Oxidation (M) | 44 | 0.002 | -1.56 |
|  |  |  |  |  |  |  | R.EAHTAASFGKPINF.- | 106 | 2.6e-009 | 0.57 |
|  |  |  |  |  |  |  | R.VCHAHPTCAEALR.E | 79 | 1.1e-006 | -5.00 |
|  |  |  |  |  |  |  | K.QELEFDVLLVSVGR.R | 69 | 1.1e-005 | -0.18 |
|  |  |  |  |  |  |  | R.MGLIGAGVIGLELGSVWGR.L + Oxidation (M) | 53 | 0.00045 | -1.63 |
|  |  |  |  |  |  |  | R.RPYTEGLGLENVGIVKDDR.G | 128 | 1.4e-011 | 2.28 |
|  |  |  |  |  |  |  | K.NKVTHLNGFGTITGPNTVVAK.M + Deamidated (NQ) | 84 | 3.4e-007 | 1.43 |
|  |  |  |  |  |  |  | K.ALLNNSHYYHMAHSGDLASR.G | 52 | 0.00058 | -4.32 |
| 24 | Q17H89 | Dihydrolipoamide succinyltransferase component of 2-oxoglutarate dehydrogenase | 9.23 | 51995 | 3 | 8 | K.LGFMSAFCK.A | 50 | 0.00099 | 11.8 |
|  |  |  |  |  |  |  | K.VPPFADSVSEGDVKFEK.K | 115 | 3.2e-010 | 7.45 |
|  |  |  |  |  |  |  | K.GQVVVRPMMYVALTYDHR.L | 24 | 0.38 | 2.60 |
| 25 | Q1HRQ7 | ATP synthase subunit alpha | 9.01 | 59526 | 11 | 24 | R.VLSIGDGIAR.V | 79 | 9.5e-007 | -23.89 |
|  |  |  |  |  |  |  | K.AVDSLVPIGR.G | 74 | 2.1e-006 | -26.58 |
|  |  |  |  |  |  |  | R.VGIKAPGIIPR.V | 24 | 0.16 | -15.02 |
|  |  |  |  |  |  |  | R.GAEISSILEER.I | 55 | 0.00033 | -17.14 |
|  |  |  |  |  |  |  | K.HALIIYDDLSK.Q | 97 | 1.8e-008 | -12.26 |
|  |  |  |  |  |  |  | R.VSVREPMQTGIK.A + Oxidation (M) | 32 | 0.062 | -14.25 |
|  |  |  |  |  |  |  | K.GIRPAINVGLSVSR.V | 26 | 0.17 | -25.00 |
|  |  |  |  |  |  |  | K.TALAIDTIINQQR.F | 81 | 7.7e-007 | -15.45 |
|  |  |  |  |  |  |  | R.EAYPGDVFYLHSR.L | 119 | 9.9e-011 | -10.79 |
|  |  |  |  |  |  |  | R.TGAIVDVPVGDELLGR.V | 63 | 4.5e-005 | -14.57 |
|  |  |  |  |  |  |  | K.NIQADEMVEFSSGLK.G + Oxidation (M) | 41 | 0.0058 | -11.58 |
| 26 | Q1HRQ7 | ATP synthase subunit alpha | 9.01 | 59526 | 11 | 28 | R.VLSIGDGIAR.V | 20 | 0.83 | -19.59 |
|  |  |  |  |  |  |  | K.AVDSLVPIGR.G | 35 | 0.019 | -18.23 |
|  |  |  |  |  |  |  | R.GAEISSILEER.I | 37 | 0.019 | -33.21 |
|  |  |  |  |  |  |  | R.VSVREPMQTGIK.A | 34 | 0.041 | -43.21 |
|  |  |  |  |  |  |  | K.GIRPAINVGLSVSR.V | 64 | 3.6e-005 | -53.67 |
|  |  |  |  |  |  |  | K.TALAIDTIINQQR.F | 88 | 1.6e-007 | -43.71 |
|  |  |  |  |  |  |  | R.EAYPGDVFYLHSR.L | 103 | 4.6e-009 | -40.98 |
|  |  |  |  |  |  |  | R.TGAIVDVPVGDELLGR.V | 79 | 1.2e-006 | -33.14 |
|  |  |  |  |  |  |  | K.NIQADEMVEFSSGLK.G | 109 | 1e-009 | -32.84 |
|  |  |  |  |  |  |  | R.EVAAFAQFGSDLDAATQQLLNR.G | 176 | 2.9e-016 | -19.41 |
|  |  |  |  |  |  |  | K.QGQYVPMAIEEQVAVIYCGVR.G | 90 | 1e-007 | -9.94 |
| 27 | Q0IG02 | 4-Hydroxybutyrate CoA-transferase, putative | 8.15 | 52102 | 5 | 15 | R.MYSGFGGQVDFIR.G | 25 | 0.29 | 118 |
|  |  |  |  |  |  |  | K.IVPTLKPGAGVVTSR.A | 30 | 0.0048 | 127 |
|  |  |  |  |  |  |  | R.AYELIQIAHPDHR.E | 69 | 7.2e-006 | 131 |
|  |  |  |  |  |  |  | R.GDAVPIFLHEIPILFR.R | 17 | 0.44 | 110 |
|  |  |  |  |  |  |  | R.AHVHYVVTENGIANLFGK.T + Deamidated (NQ) | 93 | 3e-008 | 117 |
|  | Q1HRQ7 | ATP synthase subunit alpha | 9.01 | 59526 | 3 | 7 | K.TALAIDTIINQQR.F | 17 | 0.63 | 129 |
|  |  |  |  |  |  |  | R.EAYPGDVFYLHSR.L | 32 | 0.056 | 128 |
|  |  |  |  |  |  |  | R.TGAIVDVPVGDELLGR.V | 16 | 0.76 | 126 |
| 28 | Q6QNY2 | Actin | 5.29 | 41841 | 6 | 23 | R.GYSFTTTAER.E | 63 | 4e-005 | -11.97 |
|  |  |  |  |  |  |  | R.AVFPSIVGRPR.H | 39 | 0.008 | -8.63 |
|  |  |  |  |  |  |  | K.QEYDEGGPGIVHR.K | 132 | 5.4e-012 | -9.19 |
|  |  |  |  |  |  |  | K.SYELPDGQVITIGNER.F | 121 | 7.5e-011 | -7.26 |
|  |  |  |  |  |  |  | R.VAPEEHPVLLTEAPLNPK.S | 105 | 3.1e-009 | -4.81 |
|  |  |  |  |  |  |  | K.DLYANSVLSGGTTMYPGIADR.M + Oxidation (M) | 143 | 4.7e-013 | -6.84 |
|  | Q16VS2 | Actin | 5.36 | 41835 | 5 | 20 | R.GYSFTTTAER.E | 63 | 4e-005 | -11.97 |
|  |  |  |  |  |  |  | R.AVFPSIVGRPR.H | 39 | 0.008 | -8.63 |
|  |  |  |  |  |  |  | K.SYELPDGQVITIGNER.F | 121 | 7.5e-011 | -7.26 |
|  |  |  |  |  |  |  | R.VAPEEHPILLTEAPLNPK.S | 59 | 0.00011 | -10.73 |
|  |  |  |  |  |  |  | K.DLYANSVLSGGTTMYPGIADR.M + Oxidation (M) | 143 | 4.7e-013 | -6.84 |
|  | Q17C86 | Actin | 5.30 | 42058 | 3 | 12 | K.QEYDEAGPGIVHR.K | 51 | 0.0006 | -11.59 |
|  |  |  |  |  |  |  | K.SYELPDGQVITIGNER.F | 121 | 7.5e-011 | -7.26 |
|  |  |  |  |  |  |  | R.VAPEEHPVLLTEAPLNPK.A | 105 | 3.1e-009 | -4.81 |
|  | Q17EA0 | Ubiquitin | 7.93 | 17249 | 2 | 14 | K.GSTVHLVLR.L | 55 | 0.00013 | -7.39 |
|  |  |  |  |  |  |  | K.ISEKLEEMPPNQLR.L + Oxidation (M) | 55 | 0.00033 | -5.55 |
| 29 | Q6QNY2 | Actin | 5.29 | 41841 | 7 | 26 | K.AGFAGDDAPR.A | 58 | 9.5e-005 | -17.28 |
|  |  |  |  |  |  |  | R.GYSFTTTAER.E | 55 | 0.00027 | -8.91 |
|  |  |  |  |  |  |  | R.AVFPSIVGRPR.H | 52 | 0.00039 | -6.90 |
|  |  |  |  |  |  |  | K.QEYDEGGPGIVHR.K | 122 | 5.4e-011 | -5.83 |
|  |  |  |  |  |  |  | K.SYELPDGQVITIGNER.F | 126 | 2.4e-011 | -1.53 |
|  |  |  |  |  |  |  | R.VAPEEHPVLLTEAPLNPK.S | 107 | 1.9e-009 | 2.99 |
|  |  |  |  |  |  |  | K.DLYANSVLSGGTTMYPGIADR.M + Oxidation (M) | 115 | 2.8e-010 | 3.10 |
|  | Q16VS2 | Actin | 5.36 | 41835 | 6 | 22 | K.AGFAGDDAPR.A | 58 | 9.5e-005 | -17.28 |
|  |  |  |  |  |  |  | R.GYSFTTTAER.E | 55 | 0.00027 | -8.91 |
|  |  |  |  |  |  |  | R.AVFPSIVGRPR.H | 52 | 0.00039 | -6.90 |
|  |  |  |  |  |  |  | K.SYELPDGQVITIGNER.F | 126 | 2.4e-011 | -1.53 |
|  |  |  |  |  |  |  | R.VAPEEHPILLTEAPLNPK.S | 57 | 0.00016 | -1.72 |
|  |  |  |  |  |  |  | K.DLYANSVLSGGTTMYPGIADR.M + Oxidation (M) | 115 | 2.8e-010 | 3.10 |
|  | Q17C86 | Actin | 5.30 | 42058 | 4 | 15 | K.AGFAGDDAPR.A | 58 | 9.5e-005 | -17.28 |
|  |  |  |  |  |  |  | K.QEYDEAGPGIVHR.K | 41 | 0.0069 | -7.79 |
|  |  |  |  |  |  |  | K.SYELPDGQVITIGNER.F | 126 | 2.4e-011 | -1.53 |
|  |  |  |  |  |  |  | R.VAPEEHPVLLTEAPLNPK.A | 107 | 1.9e-009 | 2.99 |
|  | Q17EA0 | Ubiquitin | 7.93 | 17249 | 2 | 14 | K.GSTVHLVLR.L | 53 | 0.00025 | -12.25 |
|  |  |  |  |  |  |  | K.ISEKLEEMPPNQLR.L + Oxidation (M) | 58 | 0.00015 | -1.04 |
| 30 | Q16P82 | Succinyl-coa synthetase beta chain | 7.68 | 48944 | 6 | 16 | R.VVFSPQEAR.D | 65 | 3.3e-005 | -10.42 |
|  |  |  |  |  |  |  | K.FGVANSAQDAEK.I | 44 | 0.0031 | -0.10 |
|  |  |  |  |  |  |  | K.MRFDDNAEFR.Q + Oxidation (M) | 54 | 0.00022 | -1.62 |
|  |  |  |  |  |  |  | R.REFYFAVMMER.A + 2 Oxidation (M) | 26 | 0.22 | -0.93 |
|  |  |  |  |  |  |  | K.MLLNMYDLFVKK.D + 2 Oxidation (M) | 22 | 0.68 | 0.36 |
|  |  |  |  |  |  |  | K.LHGGDPANFLDVGGGASVK.A | 143 | 4.8e-013 | -0.81 |
|  | Q16P83 | Succinyl-coa synthetase beta chain | 7.68 | 48642 | 6 | 16 | Matches same set of peptides | | | |
|  | Q8WQK9 | Phosphoglycerate kinase | 6.63 | 44039 | 2 | 5 | R.EAFAAPIAR.S | 41 | 0.0084 | -13.85 |
|  |  |  |  |  |  |  | K.LGDVYVNDAFGTAHR.A | 76 | 2.4e-006 | -2.34 |
|  | Q8WQL0 | Phosphoglycerate kinase | 6.63 | 44057 | 2 | 5 | Matches same set of peptides | | | |
|  | Q95UR6 | Phosphoglycerate kinase | 7.63 | 43927 | 2 | 5 | Matches same set of peptides | | | |
| 31 | Q17KK5 | Enolase | 6.28 | 46877 | 6 | 21 | K.IGSEVYHHLK.N | 45 | 0.0024 | 17.0 |
|  |  |  |  |  |  |  | K.KNGWGTMVSHR.S + Deamidated (NQ); Oxidation (M) | 61 | 7.9e-005 | 17.7 |
|  |  |  |  |  |  |  | K.DGKYDLDFKNPNSDK.S | 59 | 0.00011 | 15.0 |
|  |  |  |  |  |  |  | R.GNPTVEVDLVTDLGLFR.A | 101 | 7e-009 | 10.5 |
|  |  |  |  |  |  |  | K.VNQIGTVTESINAHLLAK.K | 88 | 1.5e-007 | 9.15 |
|  |  |  |  |  |  |  | R.AAVPSGASTGVHEALELRDNVK.A | 64 | 3.7e-005 | 11.5 |
| 32 | Q17KK5 | Enolase | 6.28 | 46877 | 7 | 25 | K.IGSEVYHHLK.N | 53 | 0.00033 | 5.83 |
|  |  |  |  |  |  |  | K.KNGWGTMVSHR.S + Deamidated (NQ); Oxidation (M) | 59 | 0.00011 | 9.39 |
|  |  |  |  |  |  |  | K.VEIGMDVAASEFHK.D + Oxidation (M) | 48 | 0.0014 | 12.4 |
|  |  |  |  |  |  |  | K.TIAPAVLNSGLCVTQQK.E | 92 | 6.6e-008 | 6.15 |
|  |  |  |  |  |  |  | R.GNPTVEVDLVTDLGLFR.A | 114 | 4e-010 | 12.4 |
|  |  |  |  |  |  |  | K.VNQIGTVTESINAHLLAK.K | 99 | 1.1e-008 | 11.3 |
|  |  |  |  |  |  |  | R.AAVPSGASTGVHEALELRDNVK.A | 78 | 1.5e-006 | 8.83 |
| 33 | Q17KK5 | Enolase | 6.28 | 46877 | 10 | 35 | K.IGSEVYHHLK.N | 34 | 0.03 | -23.77 |
|  |  |  |  |  |  |  | K.KNGWGTMVSHR.S + Deamidated (NQ); Oxidation (M) | 48 | 0.0014 | -15.33 |
|  |  |  |  |  |  |  | R.AAVPSGASTGVHEALELR.D | 94 | 3.9e-008 | -14.46 |
|  |  |  |  |  |  |  | K.TIAPAVLNSGLCVTQQK.E | 55 | 0.00028 | -10.05 |
|  |  |  |  |  |  |  | R.GNPTVEVDLVTDLGLFR.A | 132 | 6e-012 | -12.55 |
|  |  |  |  |  |  |  | K.YNQILRIEEELGSDAK.F + Deamidated (NQ) | 30 | 0.1 | -18.87 |
|  |  |  |  |  |  |  | K.VNQIGTVTESINAHLLAK.K | 102 | 5.6e-009 | -13.79 |
|  |  |  |  |  |  |  | R.AAVPSGASTGVHEALELRDNVK.A | 112 | 5.9e-010 | -17.20 |
|  |  |  |  |  |  |  | K.FGLDATAVGDEGGFAPNILENK.E | 39 | 0.012 | -6.01 |
|  |  |  |  |  |  |  | K.LAMQEFMILPTGASSFTEAMK.I + 3 Oxidation (M) | 46 | 0.0024 | -9.15 |
|  | Q8WQK9 | Phosphoglycerate kinase | 6.63 | 44039 | 2 | 5 | R.EAFAAPIAR.S | 54 | 0.00039 | -24.86 |
|  |  |  |  |  |  |  | K.LGDVYVNDAFGTAHR.A | 138 | 1.5e-012 | -19.70 |
|  | Q8WQL0 | Phosphoglycerate kinase | 6.63 | 44057 | 2 | 5 | Matches same set of peptides |  |  |  |
|  | Q95UR6 | Phosphoglycerate kinase | 7.63 | 43927 | 2 | 5 | Matches same set of peptides |  |  |  |
| 34 | Q16ZL0 | Aspartate ammonia lyase | 8.64 | 54784 | 9 | 22 | R.IESVLPR.V | 33 | 0.028 | -26.19 |
|  |  |  |  |  |  |  | K.YYGAQTVR.S | 19 | 1.1 | -10.19 |
|  |  |  |  |  |  |  | K.NCVVGIEANR.A | 32 | 0.056 | -17.55 |
|  |  |  |  |  |  |  | R.STMNFPIGGPTER.M | 61 | 8.2e-005 | -22.22 |
|  |  |  |  |  |  |  | K.VPNDKYYGAQTVR.S | 58 | 0.00016 | -26.90 |
|  |  |  |  |  |  |  | R.VYMLALGGTAVGTGLNTR.I | 62 | 6.4e-005 | -24.72 |
|  |  |  |  |  |  |  | K.SQSSNDTFPTAIHISVAR.E | 86 | 2.6e-007 | -27.81 |
|  |  |  |  |  |  |  | K.IAELTSLPFVTAPNKFEALAAR.D | 33 | 0.056 | -27.34 |
|  |  |  |  |  |  |  | R.TESDTFGELKVPNDKYYGAQTVR.S | 117 | 1.9e-010 | -23.79 |
| 35 | Q16P20 | Probable citrate synthase 2, mitochondrial | 8.91 | 51836 | 7 | 19 | R.ALGLPIERPK.S | 41 | 0.0074 | -18.66 |
|  |  |  |  |  |  |  | K.LPVVAATIYR.N | 62 | 5.1e-005 | -19.50 |
|  |  |  |  |  |  |  | K.SGQVVPGYGHAVLR.K | 96 | 2.4e-008 | -15.59 |
|  |  |  |  |  |  |  | K.VGEVTVDMMYGGMR.G + Oxidation (M) | 24 | 0.37 | 0.04 |
|  |  |  |  |  |  |  | K.EMNYYTVLFGVSR.A | 22 | 0.61 | -18.50 |
|  |  |  |  |  |  |  | K.GLVCETSVLDPDEGIR.F | 44 | 0.0041 | -15.94 |
|  |  |  |  |  |  |  | K.MLGYEDEQFTELMR.L + 2 Oxidation (M) | 32 | 0.058 | -3.96 |
|  | Q17GM7 | Probable citrate synthase 1, mitochondrial | 8.91 | 51852 | 7 | 19 | Matches same set of peptides | | | |
| 36 | Q16P20 | Probable citrate synthase 2, mitochondrial | 8.91 | 51836 | 9 | 27 | R.ALGLPIERPK.S | 32 | 0.056 | -13.38 |
|  |  |  |  |  |  |  | K.LPVVAATIYR.N | 72 | 5.8e-006 | -16.70 |
|  |  |  |  |  |  |  | K.SGQVVPGYGHAVLR.K | 95 | 2.9e-008 | -14.47 |
|  |  |  |  |  |  |  | K.VGEVTVDMMYGGMR.G | 33 | 0.049 | -13.80 |
|  |  |  |  |  |  |  | K.EMNYYTVLFGVSR.A | 55 | 0.00031 | -15.66 |
|  |  |  |  |  |  |  | K.GLVCETSVLDPDEGIR.F | 35 | 0.033 | -14.27 |
|  |  |  |  |  |  |  | K.MLGYEDEQFTELMR.L + Oxidation (M) | 54 | 0.00039 | -10.97 |
|  |  |  |  |  |  |  | K.HLPNDPLFQLVSNIYK.V | 95 | 3.4e-008 | -10.27 |
|  |  |  |  |  |  |  | K.VKNPWPNVDAHSGVLLQYYGLK.E + Deamidated (NQ) | 38 | 0.017 | -8.29 |
|  | Q17GM7 | Probable citrate synthase 1, mitochondrial | 8.91 | 51852 | 9 | 27 | Matches same set of peptides | | | |
| 37 | Q17A09 | Mitochondrial processing peptidase beta subunit | 5.87 | 52843 | 11 | 5.87 | R.GSMYWLR.V | 24 | 0.19 | -13.14 |
|  |  |  |  |  |  |  | K.APQLSPCR.F | 13 | 2.2 | -28.41 |
|  |  |  |  |  |  |  | R.EQTVFYAK.C | 25 | 0.19 | -16.36 |
|  |  |  |  |  |  |  | R.IDNVNAQNVR.D | 31 | 0.071 | -10.81 |
|  |  |  |  |  |  |  | K.LGEAEIERER.G | 36 | 0.022 | -1.17 |
|  |  |  |  |  |  |  | R.RIPLHELEKR.I | 53 | 0.00038 | -4.27 |
|  |  |  |  |  |  |  | K.VGSTFDGKAPQLSPCR.F + Deamidated (NQ) | 78 | 1.6e-006 | -0.28 |
|  |  |  |  |  |  |  | K.SDLQAYIDSHYKAPR.I | 76 | 2.1e-006 | -5.00 |
|  |  |  |  |  |  |  | R.AALVNVPPTEVTQLDSGLR.V | 100 | 9e-009 | -4.57 |
|  |  |  |  |  |  |  | R.CPAIAAVGPIENLPDYMR.I | 19 | 1.3 | -9.14 |
|  |  |  |  |  |  |  | R.VASEDSGSQTATVGLWIDAGSR.Y | 127 | 2e-011 | -5.66 |
| 38 | Q1HRN5 | Actin | 5.30 | 42164 | 7 | 26 | K.AGFAGDDAPR.A | 17 | 2 | 138 |
|  |  |  |  |  |  |  | R.GYSFTTTAER.E | 36 | 0.024 | 127 |
|  |  |  |  |  |  |  | K.QEYDESGPGIVHR.K + Deamidated (NQ) | 92 | 6.6e-008 | 105 |
|  |  |  |  |  |  |  | K.IWHHTFYNELR.V | 64 | 3.9e-005 | 105 |
|  |  |  |  |  |  |  | K.SYELPDGQVITIGNER.F | 138 | 1.5e-012 | 108 |
|  |  |  |  |  |  |  | R.VAPEEHPVLLTEAPLNPK.A | 87 | 1e-007 | 106 |
|  |  |  |  |  |  |  | K.DLYANTVMSGGTTMYPGIADR.M | 97 | 1.9e-008 | 113 |
|  | Q17KG3 | Actin | 5.22 | 42149 | 7 | 26 | K.AGFAGDDAPR.A | 17 | 2 | 138 |
|  |  |  |  |  |  |  | R.GYSFTTTAER.E | 36 | 0.024 | 127 |
|  |  |  |  |  |  |  | K.EEYDESGPGIVHR.K | 92 | 6.6e-008 | 105 |
|  |  |  |  |  |  |  | K.IWHHTFYNELR.V | 64 | 3.9e-005 | 105 |
|  |  |  |  |  |  |  | K.SYELPDGQVITIGNER.F | 138 | 1.5e-012 | 108 |
|  |  |  |  |  |  |  | R.VAPEEHPVLLTEAPLNPK.A | 87 | 1e-007 | 106 |
|  |  |  |  |  |  |  | K.DLYANTVMSGGTTMYPGIADR.M | 97 | 1.9e-008 | 113 |
|  | Q45L89 | Actin 6 | 5.23 | 42177 | 7 | 26 | Matches same set of peptides | | | |
|  | Q17C86 | Actin | 5.30 | 42058 | 5 | 17 | R.AVFPSIVGR.A | 10 | 1.7 | 148 |
|  |  |  |  |  |  |  | K.AGFAGDDAPR.A | 17 | 2 | 138 |
|  |  |  |  |  |  |  | K.IWHHTFYNELR.V | 64 | 3.9e-005 | 105 |
|  |  |  |  |  |  |  | K.SYELPDGQVITIGNER.F | 138 | 1.5e-012 | 108 |
|  |  |  |  |  |  |  | R.VAPEEHPVLLTEAPLNPK.A | 87 | 1e-007 | 106 |
| 39 | Q17AK0 | Ubiquinol-cytochrome c reductase complex core protein | 7.66 | 45884 | 6 | 19 | K.TATTFGITR.N | 49 | 0.00089 | 14.4 |
|  |  |  |  |  |  |  | R.SLNVSDADVAR.G | 70 | 9e-006 | 11.8 |
|  |  |  |  |  |  |  | K.MTVASAESGAAVAR.V | 60 | 0.00011 | 11.8 |
|  |  |  |  |  |  |  | R.HESADNLGASHVLR.N | 118 | 1.5e-010 | 16.0 |
|  |  |  |  |  |  |  | R.NLQQVGASLTATSDR.E | 98 | 1.7e-008 | 13.7 |
|  |  |  |  |  |  |  | K.KADILAAIESVSTSDVQAAAR.K | 89 | 1.3e-007 | 14.9 |
| 40 | Q16LK8 | Nadp-specific isocitrate dehydrogenase | 8.65 | 37184 | 11 | 34 | K.IWYEHR.L | 27 | 0.21 | 9.89 |
|  |  |  |  |  |  |  | R.IIWQFIK.E | 27 | 0.16 | 8.75 |
|  |  |  |  |  |  |  | K.WPLYLSTK.N | 39 | 0.011 | 10.2 |
|  |  |  |  |  |  |  | K.SDGAFVWSCK.N | 46 | 0.0021 | 13.1 |
|  |  |  |  |  |  |  | K.MWLSPNGTIR.N + Deamidated (NQ) | 60 | 0.00011 | 16.2 |
|  |  |  |  |  |  |  | R.EPILCSNIPR.L | 17 | 1.4 | 77.4 |
|  |  |  |  |  |  |  | K.KMWLSPNGTIR.N + Deamidated (NQ) | 32 | 0.065 | 16.6 |
|  |  |  |  |  |  |  | K.AQDFVIPKPGTVK.M | 59 | 0.00011 | 10.7 |
|  |  |  |  |  |  |  | R.LVPGWTRPIIIGR.H | 27 | 0.16 | 11.5 |
|  |  |  |  |  |  |  | K.CATITPDEARVEEFK.L | 32 | 0.058 | 7.56 |
|  |  |  |  |  |  |  | R.IQVAKPVVEMDGDEMTR.I | 67 | 2.1e-005 | 5.87 |
| 41 | Q178U8 | Fructose-bisphosphate aldolase | 8.45 | 39973 | 8 | 34 | K.AAQDELIKR.A | 32 | 0.059 | -15.05 |
|  |  |  |  |  |  |  | R.YASVCQSQR.I | 38 | 0.01 | -9.60 |
|  |  |  |  |  |  |  | R.FADIGVENNEDNRR.Q | 53 | 0.00052 | -7.56 |
|  |  |  |  |  |  |  | K.KPSAQEIALATVLALR.R | 156 | 2.3e-014 | -8.03 |
|  |  |  |  |  |  |  | K.NTPSYQAILENANVLAR.Y | 143 | 5e-013 | -7.34 |
|  |  |  |  |  |  |  | M.TTYFNYPPKDVQEELAR.I | 43 | 0.0057 | -13.72 |
|  |  |  |  |  |  |  | R.IVPIVEPEILPDGDHDLER.C | 126 | 2.5e-011 | -6.42 |
|  |  |  |  |  |  |  | K.GVVDLMGSEGECTTQGLDDLGAR.C | 144 | 4.2e-013 | -7.97 |
|  | Q178U9 | Fructose-bisphosphate aldolase | 8.02 | 39551 | 8 | 34 | Matches same set of peptides | | | |
| 42 | Q1HR67 | Arginine or creatine kinase | 5.97 | 40191 | 9 | 32 | R.IPFSHHDR.L | 63 | 3.7e-005 | 79.6 |
|  |  |  |  |  |  |  | R.FLQAANACR.F | 70 | 9.5e-006 | 71.4 |
|  |  |  |  |  |  |  | K.FYPLTGMDK.A | 22 | 0.45 | 78.7 |
|  |  |  |  |  |  |  | R.LVTAVNDIEKR.I | 24 | 0.31 | 45.3 |
|  |  |  |  |  |  |  | K.LESIADKYNLQVR.G | 79 | 1.2e-006 | 57.9 |
|  |  |  |  |  |  |  | R.IISMQMGGDLGQVYR.R | 77 | 1.8e-006 | 55.6 |
|  |  |  |  |  |  |  | K.AVQQQLIDDHFLFK.E | 56 | 0.00021 | 68.7 |
|  |  |  |  |  |  |  | R.LGFLTFCPTNLGTTIR.A | 75 | 3.1e-006 | 66.9 |
|  |  |  |  |  |  |  | R.GTRGEHSEAEGGIYDISNK.R | 29 | 0.14 | 82.7 |
| 43 | Q1HR67 | Arginine or creatine kinase | 5.97 | 40191 | 4 | 16 | K.LESIADKYNLQVR.G | 71 | 2.5e-006 | 135 |
|  |  |  |  |  |  |  | R.IISMQMGGDLGQVYR.R | 47 | 0.0013 | 129 |
|  |  |  |  |  |  |  | K.AVQQQLIDDHFLFK.E | 50 | 0.00052 | 125 |
|  |  |  |  |  |  |  | R.LGFLTFCPTNLGTTIR.A | 21 | 0.23 | 146 |
| 44 | Q1HR67 | Arginine or creatine kinase | 5.97 | 40191 | 9 | 30 | R.IPFSHHDR.L | 57 | 0.00011 | 145 |
|  |  |  |  |  |  |  | R.FLQAANACR.F | 65 | 2.2e-005 | 126 |
|  |  |  |  |  |  |  | R.LVTAVNDIEKR.I | 32 | 0.031 | 96.0 |
|  |  |  |  |  |  |  | K.EGDRFLQAANACR.F | 60 | 0.00011 | 93.0 |
|  |  |  |  |  |  |  | K.LESIADKYNLQVR.G | 102 | 3.6e-009 | 105 |
|  |  |  |  |  |  |  | R.IISMQMGGDLGQVYR.R | 50 | 0.00083 | 83.2 |
|  |  |  |  |  |  |  | K.AVQQQLIDDHFLFK.E | 56 | 0.00019 | 85.6 |
|  |  |  |  |  |  |  | R.LGFLTFCPTNLGTTIR.A | 69 | 1e-005 | 97.9 |
|  |  |  |  |  |  |  | R.GTRGEHSEAEGGIYDISNK.R | 66 | 2.1e-005 | 133 |
| 45 | Q03168 | Lysosomal aspartic protease | 5.22 | 42162 | 2 | 8 | K.YTGDFTYLSVDRK.A | 37 | 0.02 | 0.40 |
|  |  |  |  |  |  |  | K.QTFAEAINEPGLVFVAAK.F | 84 | 4.4e-007 | 1.69 |
| 46 | Q17H82 | Tropomyosin invertebrate | 4.80 | 32470 | 9 | 39 |  | 28 | 0.15 | 2.15 |
|  |  |  |  |  |  |  |  | 46 | 0.0025 | 1.13 |
|  |  |  |  |  |  |  |  | 36 | 0.024 | 2.44 |
|  |  |  |  |  |  |  |  | 42 | 0.0069 | 3.66 |
|  |  |  |  |  |  |  |  | 45 | 0.0029 | -14.28 |
|  |  |  |  |  |  |  |  | 51 | 0.00082 | -17.53 |
|  |  |  |  |  |  |  |  | 18 | 1.5 | -1.43 |
|  |  |  |  |  |  |  |  | 85 | 3e-007 | 0.90 |
|  |  |  |  |  |  |  |  | 31 | 0.077 | 15.8 |
| 47 | Q17KS3 | Glycerol-3-phosphate dehydrogenase | 6.47 | 38723 | 6 | 18 | R.DLFQTPNFR.V | 62 | 5.2e-005 | 17.5 |
|  |  |  |  |  |  |  | K.FVDVFYPGSK.L | 49 | 0.0013 | 21.7 |
|  |  |  |  |  |  |  | K.GMEDKFPLFTAIHR.I | 90 | 9.5e-008 | 15.7 |
|  |  |  |  |  |  |  | K.DADILIFVVPHQFIR.G | 77 | 1.9e-006 | 12.0 |
|  |  |  |  |  |  |  | K.NKGMEDKFPLFTAIHR.I | 78 | 1.6e-006 | 12.1 |
|  |  |  |  |  |  |  | R.ICTAQIKPQGFLDCLR.N | 63 | 5.1e-005 | 19.6 |
|  | Q17KS4 | Glycerol-3-phosphate dehydrogenase | 6.76 | 39837 | 6 | 18 | Matches same set of peptides | | | |
|  | Q17KS5 | Glycerol-3-phosphate dehydrogenase | 7.55 | 39447 | 6 | 18 | Matches same set of peptides | | | |
| 48 | Q17P79 | Isocitrate dehydrogenase | 8.72 | 44041 | 7 | 20 | R.MSDGLFLR.C | 67 | 1.4e-005 | 60.2 |
|  |  |  |  |  |  |  | K.LITEEASNR.V | 40 | 0.0099 | 57.5 |
|  |  |  |  |  |  |  | K.IQNACFETIR.E | 47 | 0.0018 | 51.1 |
|  |  |  |  |  |  |  | K.FGIPQSAIDSVNR.N | 97 | 2.1e-008 | 55.0 |
|  |  |  |  |  |  |  | K.EFNLYANVRPCR.S | 37 | 0.019 | 58.0 |
|  |  |  |  |  |  |  | K.IFTAANVPIEWEAVDVTPVR.N | 74 | 3.9e-006 | 36.8 |
|  |  |  |  |  |  |  | R.HMELTQHADKIQNACFETIR.E | 60 | 9.5e-005 | 44.9 |
|  | Q17P80 | Isocitrate dehydrogenase | 8.30 | 39177 | 7 | 23 | Matches same set of peptides | | | |
| 49 | Q17H82 | Tropomyosin invertebrate | 4.80 | 32470 | 9 | 39 | R.MDQLSNQLK.E | 28 | 0.15 | 2.15 |
|  |  |  |  |  |  |  | K.IMELEEELK.V | 46 | 0.0025 | 1.13 |
|  |  |  |  |  |  |  | K.IMEEVAELTKR.L | 36 | 0.024 | 2.44 |
|  |  |  |  |  |  |  | K.SLEVSEDKANQR.V | 42 | 0.0069 | 3.66 |
|  |  |  |  |  |  |  | K.LLTSTEANVAALTR.K | 45 | 0.0029 | -14.28 |
|  |  |  |  |  |  |  | K.LLEATQSADENNR.M | 51 | 0.00082 | -17.53 |
|  |  |  |  |  |  |  | R.MLAEDADGKSDEVSR.K | 18 | 1.5 | -1.43 |
|  |  |  |  |  |  |  | R.KLAFVEDELEVAEDR.V | 141 | 8.3e-013 | 5.87 |
|  |  |  |  |  |  |  | R.KVQQVEEDLEKSEER.S | 31 | 0.077 | 15.8 |
|  | Q17H80 | Tropomyosin invertebrate | 4.83 | 32616 | 9 | 39 | Matches same set of peptides | | | |
| 50 | Q16IH7 | Anterior fat body protein (AFP) | 5.54 | 34940 | 6 | 28 | R.LEAKGDIFEVR.L | 69 | 7.1e-006 | 114 |
|  |  |  |  |  |  |  | K.LDGYPVISFITPVK.G | 105 | 1.1e-009 | 112 |
|  |  |  |  |  |  |  | K.EFKTPQPPPAGALFK.I | 44 | 0.0019 | 107 |
|  |  |  |  |  |  |  | K.EYHVDENGDLSNER.K | 44 | 0.0041 | 80.0 |
|  |  |  |  |  |  |  | K.KITLIHWDGVSEQAK.F | 65 | 1.8e-005 | 106 |
|  |  |  |  |  |  |  | K.TIGEVEFDLPNNRFNDAK.T | 73 | 4.1e-006 | 102 |
| 51 | Q17D51 | Pyruvate dehydrogenase | 6.37 | 38826 | 8 | 30 | K.HITLVAHSK.A | 63 | 4.7e-005 | -13.99 |
|  |  |  |  |  |  |  | K.GVECEVINLR.S | 60 | 0.00011 | -14.03 |
|  |  |  |  |  |  |  | R.VTGVDVPMPYAK.S + Oxidation (M) | 26 | 0.24 | -5.53 |
|  |  |  |  |  |  |  | R.SLRPLDTETIFK.S | 65 | 3.4e-005 | -9.29 |
|  |  |  |  |  |  |  | K.VISPYDSEDAKGLMK.A + Oxidation (M) | 43 | 0.0052 | -6.48 |
|  |  |  |  |  |  |  | R.VFLLGEEVAQYDGAYK.V | 59 | 0.00012 | -0.67 |
|  |  |  |  |  |  |  | K.TFYMSAGTVNVPIVFR.G + Oxidation (M) | 66 | 2.2e-005 | -8.77 |
|  |  |  |  |  |  |  | K.SLEAAALPQTHDVVTAVNK.V | 84 | 4.5e-007 | -12.31 |
| 52 | Q16IA8 | Electron transport oxidoreductase (ETOa) | 8.43 | 34440 | 2 | 9 | K.FTHILAGASAFGK.A | 97 | 1.9e-008 | 19.8 |
|  |  |  |  |  |  |  | K.MLYDLADKWGAAVGASR.A + Oxidation (M) | 85 | 3.1e-007 | 21.6 |
| 53 | Q16IA8 | Electron transport oxidoreductase (ETOb) | 8.43 | 34440 | 6 | 26 | K.VKDPVKVVTVR.G | 26 | 0.13 | 18.5 |
|  |  |  |  |  |  |  | R.TIYAGNAIQTVK.V | 38 | 0.015 | 29.9 |
|  |  |  |  |  |  |  | K.FTHILAGASAFGK.A | 107 | 2e-009 | 21.5 |
|  |  |  |  |  |  |  | K.KLGGDVTVLVAGTK.V | 27 | 0.2 | -91.03 |
|  |  |  |  |  |  |  | K.MLYDLADKWGAAVGASR.A | 121 | 8.3e-011 | 16.1 |
|  |  |  |  |  |  |  | R.AAVDAGFVPNDLQIGQTGK.I | 75 | 3e-006 | 24.0 |
| 54 | Q16QZ7 | 14-3-3 protein sigma, gamma, zeta, beta/alpha | 4.73 | 29608 | 5 | 27 | K.DSTLIMQLLR.D | 37 | 0.0077 | 125 |
|  |  |  |  |  |  |  | R.YLAEFATGDDRK.D | 36 | 0.02 | 118 |
|  |  |  |  |  |  |  | K.VASMDVELTVEER.N | 47 | 0.002 | 106 |
|  |  |  |  |  |  |  | K.AASDIAMTDLPPTHPIR.L + Oxidation (M) | 70 | 8.5e-006 | 111 |
|  |  |  |  |  |  |  | R.DNLTLWTSDMQGDGDGGEQR.E | 91 | 8.6e-008 | 99.2 |
|  | Q16VF4 | Putative uncharacterized protein | 4.93 | 27681 | 3 | 17 | K.SGTCVWPDVAAR.V | 19 | 1.2 | 120 |
|  |  |  |  |  |  |  | K.ICPDGLVFDQTIR.L | 56 | 0.00019 | 105 |
|  |  |  |  |  |  |  | R.QGQCDAGLVYNEDIQR.C | 51 | 0.00083 | 101 |
| 55 | Q1HR36 | 14-3-3 protein zeta | 4.78 | 28324 | 5 | 22 | K.MKGDYYR.Y | 46 | 0.0026 | 65.8 |
|  |  |  |  |  |  |  | K.MQPTHPIR.L + Oxidation (M) | 35 | 0.025 | 51.8 |
|  |  |  |  |  |  |  | R.YLAEVATGETR.N | 78 | 1.2e-006 | 52.4 |
|  |  |  |  |  |  |  | K.SVTETGVELSNEER.N | 79 | 1.3e-006 | 51.8 |
|  |  |  |  |  |  |  | K.LAEQSERYDDMAQAMK.S | 36 | 0.023 | 45.6 |
| 56 | Q16ZI5 | Malate dehydrogenase | 9.20 | 45044 | 6 | 19 | R.VFGVSTLDIVR.A | 76 | 2.4e-006 | 29.8 |
|  |  |  |  |  |  |  | K.LNAFEQELLKK.A | 58 | 0.00014 | 11.9 |
|  |  |  |  |  |  |  | R.DDLFNTNASIVR.D | 64 | 3.6e-005 | 30.9 |
|  |  |  |  |  |  |  | K.AGAGSATLSMAYAGAR.F | 66 | 2.2e-005 | 31.4 |
|  |  |  |  |  |  |  | K.ALANADIVIIPAGVPR.K | 102 | 5.1e-009 | 31.0 |
|  |  |  |  |  |  |  | R.AMKGEQNVIECAYVR.S | 26 | 0.24 | 44.2 |
| 57 | Q17HX1 | Myosin regulatory light chain 2 (Mlc-2) | 4.63 | 22819 | 4 | 31 | K.KAPSSVFVLFSQK.Q | 33 | 0.0069 | 135 |
|  |  |  |  |  |  |  | K.AFDMNGKIDGEKFR.Y + Deamidated (NQ) | 52 | 0.00052 | 123 |
|  |  |  |  |  |  |  | R.MSGGGQDDDDVVINAFK.A | 50 | 0.00086 | 116 |
|  |  |  |  |  |  |  | K.QIAEFKEAFQLMDNDKDGVIGK.N | 31 | 0.066 | 96.8 |
| 58 | Q17HX1 | Myosin regulatory light chain 2 (Mlc-2) | 4.63 | 22819 | 4 | 31 | K.AFDMNGKIDGEKFR.Y + Deamidated (NQ) | 39 | 0.013 | 104 |
|  |  |  |  |  |  |  | K.EAFQLMDNDKDGVIGK.N | 54 | 0.00037 | 101 |
|  |  |  |  |  |  |  | R.MSGGGQDDDDVVINAFK.A + Oxidation (M) | 76 | 2.5e-006 | 101 |
|  |  |  |  |  |  |  | K.QIAEFKEAFQLMDNDKDGVIGK.N | 19 | 1.2 | 81.1 |
| 59 | Q17HX1 | Myosin regulatory light chain 2 (Mlc-2) | 4.63 | 22819 | 3 | 22 | K.AFDMNGKIDGEKFR.Y + Deamidated (NQ) | 60 | 9.2e-005 | 109 |
|  |  |  |  |  |  |  | R.MSGGGQDDDDVVINAFK.A | 85 | 3.1e-007 | 106 |
|  |  |  |  |  |  |  | K.EAFQLMDNDKDGVIGK.N | 70 | 8.1e-006 | 108 |
| 60 | Q17HX1 | Myosin regulatory light chain 2 (Mlc-2) | 4.63 | 22819 | 4 | 43 | R.MSGGGQDDDDVVINAFK.A | 79 | 8.7e-007 | -59.95 |
|  |  |  |  |  |  |  | K.EAFQLMDNDKDGVIGK.N | 67 | 1.7e-005 | -59.64 |
|  |  |  |  |  |  |  | K.ASSKEETPADTPAPAETPAAPTPSDSGSQR.G | 131 | 8.3e-012 | 4.03 |
|  |  |  |  |  |  |  | R.YALTHWGSDKFSEDEVDDAFDQMIIDDK.G | 156 | 2.4e-014 | 13.7 |
| 61 | Q17MD5 | Putative uncharacterized protein | 5.14 | 23892 | 3 | 19 | R.EYNREFLLPK.G | 49 | 0.0012 | 14.0 |
|  |  |  |  |  |  |  | K.LGDFSVIDTEFASIR.E | 127 | 1.9e-011 | 17.9 |
|  |  |  |  |  |  |  | K.LRFDVSQYAPEEIVVK.T | 76 | 2.5e-006 | 14.0 |
|  | Q17MD6 | Putative uncharacterized protein | 5.33 | 21761 | 3 | 21 | Matches same set of peptides | | | |
| 62 | Q179U9 | NADH-ubiquinone oxidoreductase 24 kDa subunit | 7.04 | 23082 | 5 | 33 | R.GAMIPLLDLAQR.Q + Oxidation (M) | 20 | 0.87 | 24.4 |
|  |  |  |  |  |  |  | R.QHGWLPISAMHR.V + Oxidation (M) | 47 | 0.0017 | 34.0 |
|  |  |  |  |  |  |  | R.VNAILNIYPEGHKR.G | 53 | 0.00046 | 22.9 |
|  |  |  |  |  |  |  | R.VYEVATFYTMFMR.K | 25 | 0.35 | 25.0 |
|  |  |  |  |  |  |  | R.KPTGTYHVQVCTTTPCWLR.G + Deamidated (NQ) | 42 | 0.0071 | 23.3 |
|  | Q1HRL6 | NADH-ubiquinone oxidoreductase 24 kDa subunit | 6.96 | 26854 | 5 | 29 | Matches same set of peptides | | | |
| 63 |  | Phosphoglycerate mutase | 6.34 | 28595 | 6 | 25 | K.FLGDEETVR.K | 37 | 0.016 | -13.14 |
|  |  |  |  |  |  |  | K.FDVAHTSLLTR.A | 93 | 4.2e-008 | -10.25 |
|  |  |  |  |  |  |  | K.NIIIAAHGNSLR.G | 57 | 0.00017 | -18.00 |
|  |  |  |  |  |  |  | K.YGEEQVLIWR.R | 84 | 3.4e-007 | -13.02 |
|  |  |  |  |  |  |  | K.EAGLKFDVAHTSLLTR.A | 60 | 9.2e-005 | -13.49 |
|  |  |  |  |  |  |  | R.YKGDPKPEEFPMAESLK.L + Oxidation (M) | 38 | 0.015 | -3.51 |
| 64 | Q177P3 | Phosphoglycerate mutase | 6.34 | 28595 | 7 | 36 | K.FLGDEETVR.K | 28 | 0.099 | -112.06 |
|  |  |  |  |  |  |  | R.HGESEWNQK.N | 59 | 2.4e-005 | -113.36 |
|  |  |  |  |  |  |  | K.FDVAHTSLLTR.A | 64 | 3.5e-005 | -117.93 |
|  |  |  |  |  |  |  | K.NIIIAAHGNSLR.G | 40 | 0.0097 | -117.12 |
|  |  |  |  |  |  |  | K.YGEEQVLIWR.R | 61 | 6.8e-005 | -114.19 |
|  |  |  |  |  |  |  | R.YKGDPKPEEFPMAESLK.L | 36 | 0.019 | -110.27 |
|  |  |  |  |  |  |  | R.SFDVPPPNMEPDHAYYDAIVKDER.Y | 46 | 0.0018 | -89.14 |
| 65 | Q17HW3 | Triosephosphate isomerase | 5.98 | 26705 | 6 | 38 | K.VIACIGETLQER.E | 51 | 0.00089 | 7.09 |
|  |  |  |  |  |  |  | R.IQYGGSVTAANCR.E | 28 | 0.15 | 7.76 |
|  |  |  |  |  |  |  | K.DLNIGWVILGHSER.R | 115 | 2.8e-010 | 3.62 |
|  |  |  |  |  |  |  | K.WFTENVSADVSGAIR.I | 145 | 2.8e-013 | 7.79 |
|  |  |  |  |  |  |  | K.TATPEQAQEVHAALRK.W | 82 | 6.1e-007 | 5.85 |
|  |  |  |  |  |  |  | R.AIFGESDELVADKVAHALAEGLK.V | 154 | 4e-014 | 2.09 |
| 66 | Q17HW3 | Triosephosphate isomerase | 5.98 | 26705 | 8 | 42 | K.FCVGGNWK.M | 61 | 6e-005 | 114 |
|  |  |  |  |  |  |  | R.EAGQTEAVCFR.Q | 91 | 6.2e-008 | 85.1 |
|  |  |  |  |  |  |  | K.VIACIGETLQER.E | 52 | 0.00058 | 86.9 |
|  |  |  |  |  |  |  | R.IQYGGSVTAANCR.E | 33 | 0.045 | 88.8 |
|  |  |  |  |  |  |  | K.DLNIGWVILGHSER.R | 96 | 1.8e-008 | 84.3 |
|  |  |  |  |  |  |  | K.WFTENVSADVSGAIR.I | 89 | 1.2e-007 | 91.4 |
|  |  |  |  |  |  |  | R.SQLPDSVGVAAQNCYK.V | 93 | 4.2e-008 | 101 |
|  |  |  |  |  |  |  | K.TATPEQAQEVHAALRK.W | 27 | 0.12 | 108 |
| 67 | Q17HW3 | Triosephosphate isomerase | 5.98 | 26705 | 5 | 26 | R.EAGQTEAVCFR.Q | 87 | 1.4e-007 | -28.47 |
|  |  |  |  |  |  |  | K.VIACIGETLQER.E | 55 | 0.00036 | -35.82 |
|  |  |  |  |  |  |  | R.IQYGGSVTAANCR.E | 64 | 3.5e-005 | -33.08 |
|  |  |  |  |  |  |  | K.DLNIGWVILGHSER.R | 89 | 7.2e-008 | 109 |
|  |  |  |  |  |  |  | K.TATPEQAQEVHAALR.K | 60 | 7e-005 | 126 |
| 68 | Q16P78 | Glutathione s-transferase | 4.98 | 23263 | 5 | 31 | K.VYYFNVK.A | 40 | 0.0095 | 43.5 |
|  |  |  |  |  |  |  | K.VHQSVAMSR.Y | 25 | 0.27 | 18.0 |
|  |  |  |  |  |  |  | K.AWIDKRPQTEI.- | 26 | 0.21 | 15.8 |
|  |  |  |  |  |  |  | R.FLLSYGNLPFDDIR.I | 120 | 1.1e-010 | 24.3 |
|  |  |  |  |  |  |  | K.LVTLNSEVIPFYLEKLDDIAR.D | 100 | 9.4e-009 | 29.2 |
| 69 | Q16P80 | Glutathione s-transferase | 5.18 | 23314 | 4 | 22 | K.VYYFNVK.A | 51 | 0.00048 | 147 |
|  |  |  |  |  |  |  | R.VHQSLAMCR.Y | 36 | 0.023 | 134 |
|  |  |  |  |  |  |  | R.FLLSYGNLPFDDIR.I | 97 | 1.3e-008 | 113 |
|  |  |  |  |  |  |  | K.TDLLTNFPQLQEVVTK.V | 74 | 1.7e-006 | 117 |
| 70 | Q1HR21 | Mitochondrial F0 ATP synthase D chain, putative | 5.26 | 19602 | 4 | 27 | K.LDWAYYQK.N | 36 | 0.02 | 49.1 |
|  |  |  |  |  |  |  | R.IADYQSQIAALK.A | 68 | 1.5e-005 | 41.2 |
|  |  |  |  |  |  |  | R.IAQSSVNWAALAER.V | 127 | 1.8e-011 | 27.0 |
|  |  |  |  |  |  |  | K.ALLPFDQMTMEDYR.D | 62 | 6.9e-005 | 37.7 |
|  | Q16P80 | Glutathione s-transferase | 5.18 | 23314 | 2 | 11 | R.VHQSLAMCR.Y | 42 | 0.006 | 28.0 |
|  |  |  |  |  |  |  | R.FLLSYGNLPFDDIR.I | 116 | 2.5e-010 | 32.2 |
| 71 | Q16W53 | Adenylate kinase isoenzyme | 5.47 | 22571 | 6 | 43 | R.APEEIFADVEK.F | 52 | 0.0006 | 37.6 |
|  |  |  |  |  |  |  | K.QGILVPNEAVLK.L | 68 | 1e-005 | 34.5 |
|  |  |  |  |  |  |  | K.ILVQYPTQLKR.I + 2 Deamidated (NQ) | 80 | 1e-006 | -7.68 |
|  |  |  |  |  |  |  | K.ALSSTVGYLIDGYPR.E | 118 | 1.4e-010 | 39.6 |
|  |  |  |  |  |  |  | R.DANVPIIWVLGGPGCGK.G | 71 | 7e-006 | 36.5 |
|  |  |  |  |  |  |  | K.YNFSHFSTGDLLREEVASGSDK.G | 53 | 0.00052 | 24.7 |
|  | Q16P80 | Glutathione s-transferase | 5.18 | 23314 | 2 | 11 | R.VHQSLAMCR.Y | 59 | 0.00012 | 31.8 |
|  |  |  |  |  |  |  | R.FLLSYGNLPFDDIR.I | 56 | 0.00026 | 32.4 |
| 72 | Q1HQT8 | Phosphatidylethanolamine-binding protein | 6.90 | 23313 | 2 | 20 | R.YVFLVYK.Q | 26 | 0.15 | 111 |
|  |  |  |  |  |  |  | R.EWHHWLVGNIPGGDVAK.G | 60 | 8.6e-005 | 71.6 |
|  |  |  |  |  |  |  | K.GETLSEYVGSGPPQGTGLHR.Y | 121 | 7.6e-011 | 80.2 |
|  | Q16LF6 | Phosphatidylethanolamine-binding protein | 6.90 | 23225 | 3 | 20 | Matches same set of peptides | | | |
|  | Q6QNY2 | Actin | 5.29 | 41841 | 4 | 13 | R.GYSFTTTAER.E | 11 | 7.7 | 102 |
|  |  |  |  |  |  |  | R.AVFPSIVGRPR.H | 21 | 0.25 | 92.4 |
|  |  |  |  |  |  |  | K.QEYDEGGPGIVHR.K | 41 | 0.008 | 84.4 |
|  |  |  |  |  |  |  | K.SYELPDGQVITIGNER.F | 88 | 1.7e-007 | 74.7 |
|  | Q16W51 | Adenylate kinase isoenzyme (Fragment) | 6.95 | 22975 | 3 | 19 | K.ILVQYPTQLKR.V | 32 | 0.024 | 81.3 |
|  |  |  |  |  |  |  | K.YNFSHFSTGDLLR.E | 44 | 0.0041 | 81.3 |
|  |  |  |  |  |  |  | K.ALSSTVGYLIDGYPR.E | 55 | 0.00025 | 75.6 |
|  | Q16W52 | Adenylate kinase isoenzyme | 7.66 | 20698 | 3 | 21 | Matches same set of peptides | | | |
|  | Q16W53 | Adenylate kinase isoenzyme | 5.47 | 22571 | 3 | 19 | Matches same set of peptides | | | |
| 73 | Q17BT9 | Superoxide dismutase (SOD2a) | 8.35 | 24618 | 6 | 38 | K.IIQLGGALK.F | 63 | 1.5e-005 | 10.4 |
|  |  |  |  |  |  |  | R.EIMEVHHQK.H | 63 | 4.1e-005 | 7.43 |
|  |  |  |  |  |  |  | K.FNGGGHINHSIFWK.N + Deamidated (NQ) | 104 | 4e-009 | 2.28 |
|  |  |  |  |  |  |  | K.LLERDFHGLENFKK.E | 33 | 0.055 | 4.87 |
|  |  |  |  |  |  |  | K.NLRPNYVDAIWDVVNWK.D | 41 | 0.0087 | 1.84 |
|  |  |  |  |  |  |  | K.HHNAYVTNLNAAEEQLAEAVAK.K | 107 | 2.1e-009 | -6.04 |
| 74 | Q17BT9 | Superoxide dismutase (SOD2b) | 8.35 | 24618 | 3 | 20 | K.FNGGGHINHSIFWK.N | 103 | 4.6e-009 | 29.6 |
|  |  |  |  |  |  |  | K.LLERDFHGLENFKK.E | 27 | 0.21 | 19.9 |
|  |  |  |  |  |  |  | K.NLRPNYVDAIWDVVNWK.D | 39 | 0.013 | 16.3 |
| 75 | Q16XK7 | Calcium-binding protein, putative | 4.68 | 22124 | 5 | 56 | R.YQELYAQFMGNESDK.C | 129 | 7.7e-012 | -73.49 |
|  |  |  |  |  |  |  | K.KVDDSYNQLVSDEDNKR.G | 71 | 8.5e-006 | -52.91 |
|  |  |  |  |  |  |  | K.ALWDEISALADLDHDGKITTEEFK.D | 64 | 3.5e-005 | -22.22 |
|  |  |  |  |  |  |  | K.AFIDAHYQMMDINNDGLVSIEEYR.Y | 205 | 3.2e-019 | 17.9 |
|  |  |  |  |  |  |  | R.VEFIVGHMYDIDNNGFLDNNDFMCMALR.A | 70 | 7.8e-006 | 31.0 |
| 76 | Q1HQI1 | Mitochondrial NADH:ubiquinone oxidoreductase NDUFA8/PGIV/19 kDa subunit (Fragment) | 6.44 | 20345 | 4 | 32 | K.AVTNCALEFFR.K | 67 | 1.9e-005 | 1.26 |
|  |  |  |  |  |  |  | K.SSGDLRFEYCR.K | 20 | 0.89 | 5.63 |
|  |  |  |  |  |  |  | K.ACEAENNEFMLCR.Q | 81 | 6.7e-007 | 0.19 |
|  |  |  |  |  |  |  | K.TVYPDATPGLPEDYPRPDAK.Y | 62 | 6.7e-005 | 0.69 |
| 77 | Q17I02 | ATP synthase delta chain, mitochondrial | 5.23 | 17020 | 4 | 41 | K.VYYDSAHIR.Q | 69 | 1.1e-005 | 58.3 |
|  |  |  |  |  |  |  | R.QVDVPSFSGAFGILPK.H | 103 | 5.2e-009 | 55.0 |
|  |  |  |  |  |  |  | R.EILSSAQSQLSSASTDKER.A | 196 | 2.4e-018 | 48.7 |
|  |  |  |  |  |  |  | K.HVPTLAVLKPGVVTVYEQDGATK.K | 127 | 1.8e-011 | 48.6 |
|  | Q17I03 | ATP synthase delta chain, mitochondrial | 5.40 | 18495 | 4 | 41 | Matches same set of peptides | | | |
| 78 | Q1HRK3 | Transcription factor BTF3A | 6.32 | 17102 | 4 | 44 | K.NDGTVIHFNNPK.T | 57 | 0.00011 | 132 |
|  |  |  |  |  |  |  | K.LGVNTIPGIEEVNMIK.N + Oxidation (M) | 18 | 0.92 | 107 |
|  |  |  |  |  |  |  | K.TQASLATNTFAITGHSESK.Q | 146 | 2.3e-013 | 112 |
|  |  |  |  |  |  |  | K.QITDMLPSIITQLGPEGLNQLKK.L | 53 | 0.00034 | 69.2 |
| 79 | Q1HRP6 | Peptidyl-prolyl cis-trans isomerase | 8.71 | 21435 | 3 | 20 | K.QTSWLDGR.H | 8 | 13 | -24.49 |
|  |  |  |  |  |  |  | R.VIQDFMIQGGDFTR.G + Deamidated (NQ); Oxidation (M) | 59 | 0.00011 | -7.23 |
|  |  |  |  |  |  |  | K.VTDKVFFDITIGGKPEGR.I | 104 | 3.4e-009 | -13.80 |
| 80 | Q175S9 | Troponin C | 3.92 | 17579 | 3 | 17 | K.GFIPVDSFK.Q | 45 | 0.0023 | -1.60 |
|  |  |  |  |  |  |  | R.EVFMMYDK.E | 50 | 0.00084 | -16.66 |
|  |  |  |  |  |  |  | K.EAFDAFDKDK.T | 63 | 4.1e-005 | 0.76 |
|  | Q175T1 | Troponin C | 3.91 | 17862 | 3 | 17 | Matches same set of peptides | | | |
| 81 | Q1HRN6 | Initiation factor 5a (Fragment) (eIF5A) | 5.30 | 17855 | 3 | 27 | K.IPDGDLGTQLR.S | 57 | 0.00018 | 7.20 |
|  |  |  |  |  |  |  | R.SEFDSGKELVCTVLK.S | 117 | 1.7e-010 | 14.4 |
|  |  |  |  |  |  |  | K.KYEDICPSTHNMDVPHVK.R | 44 | 0.0044 | -1.39 |
|  | Q6QNY2 | Actin | 5.29 | 41841 | 8 | 28 | K.AGFAGDDAPR.A | 34 | 0.04 | 13.4 |
|  |  |  |  |  |  |  | K.IKIIAPPER.K | 32 | 0.044 | -4.49 |
|  |  |  |  |  |  |  | R.AVFPSIVGRPR.H | 50 | 0.00065 | 16.2 |
|  |  |  |  |  |  |  | K.QEYDEGGPGIVHR.K | 49 | 0.0014 | 7.64 |
|  |  |  |  |  |  |  | K.IWHHTFYNELR.V | 18 | 1.7 | -0.80 |
|  |  |  |  |  |  |  | K.SYELPDGQVITIGNER.F | 130 | 1.1e-011 | 2.99 |
|  |  |  |  |  |  |  | R.VAPEEHPVLLTEAPLNPK.S | 101 | 7.9e-009 | 9.67 |
|  |  |  |  |  |  |  | K.DLYANSVLSGGTTMYPGIADR.M | 79 | 1.4e-006 | -2.11 |
|  | Q178A9 | Actin | 5.29 | 41958 | 7 | 25 | K.AGFAGDDAPR.A | 34 | 0.04 | 13.4 |
|  |  |  |  |  |  |  | K.IKIIAPPER.K | 32 | 0.044 | -4.49 |
|  |  |  |  |  |  |  | R.AVFPSIVGRPR.H | 50 | 0.00065 | 16.2 |
|  |  |  |  |  |  |  | K.IWHHTFYNELR.V | 18 | 1.7 | -0.80 |
|  |  |  |  |  |  |  | K.SYELPDGQVITIGNER.F | 130 | 1.1e-011 | 2.99 |
|  |  |  |  |  |  |  | R.VAPEEHPVLLTEAPLNPK.A | 101 | 7.9e-009 | 9.67 |
|  |  |  |  |  |  |  | K.DLYANTVLSGGTTMYPGIADR.M | 58 | 0.00015 | -10.45 |
|  | P49128 | Actin-1 | 5.30 | 42045 | 7 | 25 | Matches same set of peptides | | | |
|  | Q4PKE5 | Actin 5 | 5.30 | 42194 | 7 | 25 | Matches same set of peptides | | | |
|  | Q16QR7 | Actin | 5.30 | 42194 | 7 | 25 | Matches same set of peptides | | | |
| 82 | Q17J75 | Cuticle protein, putative | 6.82 | 17504 | 3 | 35 | R.SGDVVTGSYSLVEPDGTR.R | 49 | 0.0011 | -7.14 |
|  |  |  |  |  |  |  | K.VAAPLAYPAAHVGYPAYGK.A | 105 | 3.1e-009 | -7.11 |
|  |  |  |  |  |  |  | R.VVEYTADPVNGFNAVVHREPLAVK.A + Deamidated (NQ) | 57 | 0.00017 | 3.87 |
| 83 | Q17JZ6 | NADH dehydrogenase, putative | 6.59 | 19643 | 3 | 19 | K.AAENWFIK.Y | 36 | 0.023 | -11.86 |
|  |  |  |  |  |  |  | K.YGDLGGYANAK.T | 51 | 0.00042 | -16.16 |
|  |  |  |  |  |  |  | R.DKMVDNEILNILR.Q + Oxidation (M) | 73 | 5.5e-006 | -15.66 |
| 84 | Q17J75 | Cuticle protein, putative | 6.82 | 17504 | 3 | 19 | R.SGDVVTGSYSLVEPDGTR.R | 28 | 0.17 | 0.02 |
|  |  |  |  |  |  |  | K.VAAPLAYPAAHVGYPAYGK.A | 67 | 2e-005 | 0.15 |
|  |  |  |  |  |  |  | R.VVEYTADPVNGFNAVVHREPLAVK.A + Deamidated (NQ) | 39 | 0.011 | -8.90 |
| 85 | Q1HQJ3 | Mitochondrial NADH:ubiquinone oxidoreductase NDUFS4/18 kDa subunit | 9.33 | 21252 | 4 | 31 | R.WFVDNPEVVKK.H | 27 | 0.21 | -5.57 |
|  |  |  |  |  |  |  | R.VKNYGVNFAWNKR.T | 76 | 2.7e-006 | -10.05 |
|  |  |  |  |  |  |  | K.EAPIVDANLVIADEVER.R | 69 | 1.3e-005 | -11.34 |
|  |  |  |  |  |  |  | K.VDLSPISGVPEEHVKER.R | 37 | 0.019 | -11.52 |
| 86 | Q1HQF5 | Actin depolymerizing factor | 6.74 | 17323 | 7 | 60 | K.QIDVEVIGDR.N | 56 | 0.00023 | 5.82 |
|  |  |  |  |  |  |  | R.YVIFYIRDEK.Q | 45 | 0.0032 | 4.52 |
|  |  |  |  |  |  |  | K.YIQATDLSEASR.E | 94 | 3.6e-008 | 6.66 |
|  |  |  |  |  |  |  | K.MLYSSSFDALKK.S | 81 | 8.5e-007 | 15.4 |
|  |  |  |  |  |  |  | K.LFLMSWCPDTAK.V | 101 | 6.7e-009 | 5.75 |
|  |  |  |  |  |  |  | R.NAEYDQFLEDIQK.G | 112 | 6.5e-010 | 10.5 |
|  |  |  |  |  |  |  | R.YGLFDFEYMHQCQGTSESSK.K | 74 | 3.4e-006 | -3.76 |
|  | Q17EA0 | Ubiquitin | 7.93 | 17249 | 3 | 25 | K.GSTVHLVLR.L | 20 | 0.53 | 13.0 |
|  |  |  |  |  |  |  | K.ISEKLEEMPPNQLR.L | 51 | 0.00073 | 6.96 |
|  |  |  |  |  |  |  | R.TMAIDTEPEDSVETLR.V | 68 | 1.7e-005 | -0.96 |
|  | Q1HRN5 | Actin | 5.30 | 42164 | 2 | 7 | R.AVFPSIVGRPR.H | 54 | 0.00027 | 15.1 |
|  |  |  |  |  |  |  | K.SYELPDGQVITIGNER.F + 2 Deamidated (NQ) | 37 | 0.022 | 49.9 |
|  | Q4PKE5 | Actin 5 | 5.30 | 42194 | 2 | 7 | Matches same set of peptides | | | |
|  | Q6ELZ6 | Muscle-specific actin 3 | 5.44 | 41934 | 2 | 7 | Matches same set of peptides | | | |
|  | Q6QNY2 | Actin | 5.29 | 41841 | 2 | 7 | Matches same set of peptides | | | |
|  | Q16QR7 | Actin | 5.30 | 42194 | 2 | 7 | Matches same set of peptides | | | |
|  | Q16VS2 | Actin | 5.36 | 41835 | 2 | 7 | Matches same set of peptides | | | |
|  | Q17C87 | Actin | 5.29 | 42160 | 2 | 7 | Matches same set of peptides | | | |
|  | Q17KG3 | Actin | 5.22 | 42149 | 2 | 7 | Matches same set of peptides | | | |
|  | Q45L89 | Actin 6 | 5.23 | 42177 | 2 | 7 | Matches same set of peptides | | | |
|  | Q178A9 | Actin | 5.29 | 41958 | 2 | 7 | Matches same set of peptides | | | |
|  | P49128 | Actin-1 | 5.30 | 42045 | 2 | 7 | Matches same set of peptides | | | |
| 87 | Q1HQF5 | Actin depolymerizing factor (ADF) | 6.74 | 17323 | 4 | 36 | R.YVIFYIRDEK.Q | 36 | 0.023 | 2.97 |
|  |  |  |  |  |  |  | K.YIQATDLSEASR.E | 58 | 0.00016 | 2.13 |
|  |  |  |  |  |  |  | K.LFLMSWCPDTAK.V | 68 | 1.5e-005 | 7.26 |
|  |  |  |  |  |  |  | R.YGLFDFEYMHQCQGTSESSK.K | 77 | 1.7e-006 | 2.80 |
| 88 | Q16MS5 | Myosin light chain 1, putative | 4.40 | 18147 | 3 | 31 | R.ALNLNPTLELIGK.M + Deamidated (NQ) | 72 | 4.4e-006 | -24.02 |
|  |  |  |  |  |  |  | R.IKLEEFLPIYAQVK.K | 109 | 9.9e-010 | -5.54 |
|  |  |  |  |  |  |  | K.DCMDPEDDDGNIPYAPFLQR.L + Oxidation (M) | 34 | 0.018 | -24.58 |
| 89 | Q1HRJ0 | Cytochrome c oxidase polypeptide | 5.39 | 17135 | 3 | 17 | R.YEAYFGR.S | 44 | 0.0035 | 2.91 |
|  |  |  |  |  |  |  | R.LNDYALAIR.F | 46 | 0.0023 | -6.28 |
|  |  |  |  |  |  |  | R.SDIDHWEAR.K | 36 | 0.017 | -17.24 |
| 90 | Q1HRN3 | Superoxide dismutase [Cu-Zn] | 5.77 | 15616 | 5 | 53 | R.LACGVIGICKA.- | 29 | 0.072 | 138 |
|  |  |  |  |  |  |  | R.HAGDLGNVVADGSGVAK.V | 100 | 6.9e-009 | 108 |
|  |  |  |  |  |  |  | K.GTIFFQQNGDSDPVK.V + Deamidated (NQ) | 66 | 2.2e-005 | 108 |
|  |  |  |  |  |  |  | K.VDISDSQISLSGPLSILGR.T | 119 | 7.4e-011 | 102 |
|  |  |  |  |  |  |  | R.TVVVHADPDDLGLGGHELSK.S | 90 | 7.5e-008 | 99.4 |
| 91 | Q17LN8 | Putative uncharacterized protein (Q17LN8) | 9.02 | 25401 | 2 | 11 | K.AQHFAAHAAAR.A | 72 | 3.3e-006 | 128 |
|  |  |  |  |  |  |  | R.GLVGAGPIDTPEVQHAK.A | 32 | 0.044 | 92.8 |
|  | Q5UG08 | Peroxiredoxin-like protein | 6.71 | 16862 | 2 | 15 | K.THLPGYVDR.A | 11 | 4.8 | 131 |
|  |  |  |  |  |  |  | K.QLELGADLPPLGGLR.S | 60 | 4.7e-005 | 94.6 |
|  | Q173J1 | Peroxiredoxin 5, prdx5 | 9.05 | 20918 | 2 | 12 | Matches same set of peptides | | | |
| 92 | Q5UG08 | Peroxiredoxin-like protein | 6.71 | 16862 | 3 | 25 | K.THLPGYVDR.A | 36 | 0.02 | 15.1 |
|  |  |  |  |  |  |  | K.QLELGADLPPLGGLR.S | 58 | 0.00016 | 8.22 |
|  |  |  |  |  |  |  | K.VVLFAVPGAFTPGCSK.T | 58 | 0.00015 | 13.0 |
|  | Q173J1 | Peroxiredoxin 5, prdx5 | 9.05 | 20918 | 3 | 20 | Matches same set of peptides | | | |
| 93 | Q177Y4 | Allergen, putative | 6.62 | 14908 | 2 | 25 | R.EFTDTDLIATMTAGNAK.C | 46 | 0.0024 | 4.93 |
|  |  |  |  |  |  |  | K.FKLGEEFEEETVDGRK.V | 84 | 3.6e-007 | 7.82 |
| 94 | Q1HRL7 | Odorant binding protein | 5.63 | 16198 | 3 | 34 | K.WEFPEDDTTMCYIK.C | 61 | 7.9e-005 | 73.8 |
|  |  |  |  |  |  |  | K.CVDKNTDNNACHWAFR.G | 100 | 1e-008 | 83.3 |
|  |  |  |  |  |  |  | R.YRTECVSSLNIPADYVEK.F | 83 | 5.2e-007 | 97.4 |
| 95 | Q16KF9 | Cytochrome c oxidase, subunit VB, putative | 8.59 | 14013 | 3 | 27 | R.QAGDNDPFDMR.V | 45 | 0.0031 | 82.8 |
|  |  |  |  |  |  |  | K.RCECGHWFK.L | 25 | 0.31 | 93.3 |
|  |  |  |  |  |  |  | K.ESPNMIPSAFESR.L + Oxidation (M) | 65 | 3.2e-005 | 112 |
| 96 | Q5MM88 | Ubiquinol-cytochrome C reductase binding protein | 6.74 | 13368 | 2 | 29 | R.WAYNLSGFNQYGLHR.D | 92 | 6.3e-008 | 2.24 |
|  |  |  |  |  |  |  | K.YEEDCKYLEPYLEEVKR.E | 77 | 1.8e-006 | 1.78 |
