# Supplementary material for: Proteomic Biomarkers for Ageing the Mosquito Aedes aegypti to Determine Risk of Pathogen Transmission
Source: PLoS One. 2013 Mar 11;8(3):e58656. doi: 10.1371/journal.pone.0058656 (PMC3594161; doi:10.1371/journal.pone.0058656)
Supplement: Table S4 — Custom prepared polyclonal antibodies and Western analysis conditions used to validate Ae. aegypti ageing biomarker proteins. (DOCX) [file pone.0058656.s010.docx]

| **Primary antibody** | **Type** | **Antigen**  **(Tag-Uniprot accession; coverage)** | **Dilution** | **Secondary antibody** | **Dilution** |
| --- | --- | --- | --- | --- | --- |
| SOD2_pan | Rabbit pAb | GST-Q17BT9; aa 1-222 | 1:1000 | Goat α rabbit IR800CW (Li-cor) | 1:20000 |
| ETO_pan | Rabbit pAb | GST-Q16IA8; aa 1-330 | 1:1000 | Goat α rabbit IR800CW (Li-cor) | 1:20000 |
| ADF | Rabbit pAb | GST-A0NGL9; aa 1-153 | 1:1000 | Goat α rabbit IR800CW (Li-cor) | 1:20000 |
| eIF5A^†^ | Rabbit sera | HIS-C6ZJP8 | 1:5000 | Goat α rabbit IR800CW (Li-cor) | 1:20000 |
| Q17LN8 | Sheep pAb | GST-Q17LN8; aa 84-120 | 1:250 | Donkey α goat 800CW (Li-Cor) | 1:10000 |
| AFP | Rabbit pAb | GST-Q16IH7; aa 1-78 | 1:500 | Goat α rabbit IR800CW (Li-cor) | 1:20000 |

† Rabbit α eIF5A sera provided by Dr Wei-June Chen, Chang Gung University, Taiwan
